# Approximation of state variables for discrete-time stochastic genetic regulatory networks with leakage, distributed, and probabilistic measurement delays: a robust stability problem

**DOI:** 10.1186/s13662-018-1569-z

**Published:** 2018-04-03

**Authors:** S. Pandiselvi, R. Raja, Jinde Cao, G. Rajchakit, Bashir Ahmad

**Affiliations:** 1 Department of Mathematics, Alagappa University, Karaikudi, India; 2 Ramanujan Centre for Higher Mathematics, Alagappa University, Karaikudi, India; 3 School of Mathematics, Southeast University, Nanjing, China; 4 Department of Mathematics, Faculty of Science, Maejo University, Chiang Mai, Thailand; 5 Nonlinear Analysis and Applied Mathematics (NAAM) Research Group, Department of Mathematics, Faculty of Science, King Abdulaziz University, Jeddah, Saudi Arabia

**Keywords:** Genetic regulatory networks (GRNs), Time-varying delays, Distributed delays, Leakage delays, Probabilistic measurement delays

## Abstract

This work predominantly labels the problem of approximation of state variables for discrete-time stochastic genetic regulatory networks with leakage, distributed, and probabilistic measurement delays. Here we design a linear estimator in such a way that the absorption of mRNA and protein can be approximated via known measurement outputs. By utilizing a Lyapunov–Krasovskii functional and some stochastic analysis execution, we obtain the stability formula of the estimation error systems in the structure of linear matrix inequalities under which the estimation error dynamics is robustly exponentially stable. Further, the obtained conditions (in the form of LMIs) can be effortlessly solved by some available software packages. Moreover, the specific expression of the desired estimator is also shown in the main section. Finally, two mathematical illustrative examples are accorded to show the advantage of the proposed conceptual results.

## Introduction and system formulation

A gene is a physical structure made up of DNA, and most of the genes hold the data which is required to make molecules called as proteins. In the modern years, research in genetic regulatory networks (GRNs) has gained significance in both biological and bio-medical sciences, and a huge number of tremendous results have been issued. Distinct kinds of computational models have been applied to propagate the behaviors of GRNs; see, for instance, the Bayesian network models, the Petri net models, the Boolean models, and the differential equation models. Surrounded by the indicated models, the differential equation models describe the rate of change in the concentration of gene production, such as mRNAs and proteins, as constant values, whereas the other models do not have such a basis.

As one of the mostly investigated dynamical behaviors, the state estimation for GRNs has newly stirred increasing research interest (see [1, 2] and the references cited therein [1, 3–10]). In fact, this is an immense concern since GRNs are complex nonlinear systems. Due to the complication, it is frequently the case that only partial facts around the states of the nodes are accessible in the network outputs. In consideration of realizing the GRNs better, there has been a necessity to estimate the state of the nodes through securable measurements. In [1], the robust $H_{\infty}$ problem was considered for a discrete-time stochastic GRNs with probabilistic measurement delays. In [2], the robust $H_{\infty}$ state estimation problem was investigated for a general class of uncertain discrete-time stochastic neural networks with probabilistic measurement delays. By designing an adaptive controller, the authors investigated the problem of delayed GRNs stabilization in [7]. Xiao et al. discussed the stability, periodic oscillation, and bifurcation of two-gene regulatory networks with time delays [8]. The stability of continuous GRNs and discrete-time GRNs was discussed, respectively, in [11]. Huang et al. considered the bifurcation of delayed fractional GRNs by hybrid control [12].

Due to the limited signal communication speed, the measurement among the networks is always assumed to be a delayed one. So, the network measurement could not include instruction about the present gene states, while the delayed network measurement could. The most fashionable mechanism to relate the probabilistic measurement delay or some other kind of lacking measurement is to grab it as a Bernoulli distributed white classification [13–20]. The robust stochastic stability of stochastic genetic GRNs was considered, and some delay-dependent criteria were presented in the form of LMIs [18]. And the asymptotic stability of delayed stochastic GRNs with impulsive effect was discussed in [19]. The synchronization problem of dynamical system was also discussed in [21, 22]. The challenging task is how to draft the robust estimators when both uncertainties and probabilistic appeared in discrete-time GRN models.

More recently, in [23], Liu et al. developed a state estimation problem for a genetic regulatory network with Markovian jumping parameters and time delays:

$$\begin{gathered} \dot{m}(t)=-A\bigl(r(t)\bigr)m(t)+W\bigl(r(t)\bigr)g\bigl(p\bigl(t-\sigma(t)\bigr)\bigr), \\ \dot{p}(t)=-C\bigl(r(t)\bigr)p(t)+D\bigl(r(t)\bigr)m\bigl(t-\tau(t)\bigr).\end{gathered} $$

Also in [24], Wan et al. proposed the state estimation of discrete-time GRN with random delays governed by the following equation:

$$\begin{gathered} M(k+1)=AM(k)+B f\bigl(P\bigl(k-d(k)\bigr)\bigr)+V, \\ P(k+1)=CP(k)+DM\bigl(k-\tau(k)\bigr).\end{gathered} $$

Considering the above referenced papers, the robustness of approximation of the stochastic GRNs with leakage delays, distributed delays, and probabilistic measurement delays has not been tackled. The main contributions of this paper are summarized as follows:

1. We examine the approximation concern for the discrete-time stochastic GRNs with the leakage delays, distributed delays, and probabilistic measurement delays into the problem and model the robust $H_{\infty}$ state estimator for a class of discrete-time stochastic GRNs. Here, the probabilistic measurement delays, which narrate the binary shifting sequence, are satisfied by the conditional probability distribution. So, the crisis of parameter uncertainties, including errors, stochastic disturbance, leakage delays, distributed delays, and the activation function of the addressed GRNs, is identified by sector-bounded nonlinearities.
2. By applying the Lyapunov stability theory and stochastic analysis techniques, sufficient conditions are first entrenched to assure the presence of the desired estimators in terms of a linear matrix inequality (LMI). These circumstances are reliant on both the lower and upper bounds of time-varying delays. Again, the absolute expression of the desired estimator is demonstrated to assure the estimation error dynamics to be robustly exponentially stable in the mean square for the consigned system.
3. Finally, twin mathematical examples beside with simulations are given to view the capability of the advanced criteria.

In this note, we consider the GRNs with leakage, discrete, and distributed delays described as follows:

1 $$\begin{aligned}& \begin{aligned}x(k+1)={}&{-}\bigl(\mathbb{A}+\Delta\mathbb{A}(k)\bigr)x(k-\rho_{1})+ \bigl(\mathbb {B}+\Delta\mathbb{B}(k)\bigr) \hat{g}\bigl(y\bigl(k-\delta(k)\bigr) \bigr) \\ &+\bigl(E+\Delta E(k)\bigr)\sum_{s=1}^{\infty} \mu_{s}h\bigl(y(k-s)\bigr)+\sigma \bigl(k,x(k-\rho_{1}) \bigr)\omega(k)+L_{x}v_{x}(k),\end{aligned} \\& \begin{aligned}[b] y(k+1)={}&{-}\bigl(\mathbb{C}+\Delta\mathbb{C}(k)\bigr)y(k-\rho_{2})+ \bigl(\mathbb {D}+\Delta\mathbb{D}(k)\bigr)x\bigl(k-\tau(k)\bigr) \\ &+\bigl(F+\Delta F(k)\bigr)\sum_{n=1}^{\infty} \xi _{n}x(k-n)+L_{y}v_{y}(k), \end{aligned} \end{aligned}$$

where $x(k-\rho_{1})=[x_{1}(k-\rho_{1}), \ldots, x_{n}(k-\rho _{2})]^{T}\in\mathbb{R}^{n}$, $y(k-\rho_{2})=[y_{1}(k-\rho_{2}), \ldots, y_{n}(k-\rho_{2})]^{T}\in \mathbb{R}^{n}$, $x_{i}(k-\rho_{1})$, and $y_{i}(k-\rho_{2})$ ($i=1,2,\ldots,n$) denote the concentrations of mRNA and protein of the *i*th node at time *t*, respectively; $\mathbb{A}=\operatorname{diag}\{a_{1}, a_{2}, \ldots, a_{n}\}$, $\mathbb{C}=\operatorname{diag}\{c_{1}, c_{2}, \ldots, c_{n}\}$, and $\mathbb{D}=\operatorname{diag}\{d_{1}, d_{2}, \ldots, d_{n}\}$ are constant matrices; $a_{i}>0$, $c_{i}>0$, and $d_{i}>0$ are the degradation rates of mRNAs, protein, and the translation rate of the *i*th gene, respectively; the coupling matrix of the genetic regulatory network is defined as $\mathbb{B}=(b_{ij})\in\mathbb{R}^{n\times n}$; $E=\operatorname{diag}\{e_{1}, e_{2}, \ldots, e_{n}\}$, and $F=\operatorname {diag}\{f_{1}, f_{2}, \ldots, f_{n}\}$ are the weight matrices. $\Delta \mathbb{A}(k)$, $\Delta\mathbb{B}(k)$, $\Delta\mathbb{C}(k)$, $\Delta\mathbb{D}(k)$, $\Delta\mathbb{E}(k)$, and $\Delta\mathbb {F}(k)$ represent the parameter uncertainties; $h(y(k))=[h_{1}(y(k)), \ldots, h_{n}(y(k))]^{T}\in\mathbb{R}^{n}$ denotes the activation function; the exogenous disturbance signals $v_{x}(k), v_{y}(k)\in \mathbb{R}^{n}$ satisfy $v_{i}(\cdot)\in L_{2} [0,\infty)$. $L_{x}$ and $L_{y}$ are the known real constant matrices. $\delta(k)$ denotes the feedback regulation delay and $\tau(k)$ denotes the translation delay, which satisfy

2 $$ 0\leq\delta_{m}\leq\delta(k)\leq\delta_{M},\qquad 0\leq \tau_{m}\leq \tau(k)\leq\tau_{M}, $$

where the lower bound $\delta_{m}$, $\tau_{m}$ and the upper bound $\delta_{M}$, $\tau_{M}$ are known positive integers.

Furthermore, the nonlinear activation function $\hat{g}(y(k-\delta (k)))=[\hat{g}_{1}(y_{1}(k-\delta(k))),\ldots, \hat {g}_{n}(y_{n}(k-\delta(k)))]^{T}\in\mathbb{R}^{n}$ represents the feedback regulation of the protein on the transcription. It is a monotonic function in the Hill form, that is, $\hat{g}_{i}(f)=\frac{f^{h_{j}}}{1+f^{h_{j}}}$ ($j=1,2,\ldots,n$), where $h_{j}$ is the Hill co-efficient and *f* is a positive constant. The noise intensity function vector $\sigma(k,x(k)):\mathbb{R}\times \mathbb{R}^{n}\rightarrow\mathbb{R}^{n}$ satisfies

3 $$ \sigma^{T}\bigl(k,x(k-\rho_{1})\bigr)\sigma\bigl(k,x(k- \rho_{1})\bigr)\leq x^{T}(k-\rho _{1})H x(k- \rho_{1}), $$

where $H>0$ is a known matrix. $\omega(k)$ is a Brownian motion with $\mathbb{E}\{\omega(k)\}=0$, $\mathbb{E}\{\omega^{2}(k)\}=1$ and $\mathbb{E}\{\omega(i)\omega(j)\}=0$ ($i\neq j$).

For large-scale complex networks, information around the network nodes is not often fully attainable from the network outputs (see [25, 26]). We can assume that network measurements are described as follows:

4 $$\begin{aligned}& Z_{x}(k)=M x(k), \\& Z_{y}(k)=N y(k), \end{aligned}$$

where *M* and *N* are known constant matrices. $Z_{x}(k), Z_{y}(k)\in \mathbb{R}^{l}$ are the complete outputs of the network. The network outputs are subjected to probabilistic delays that can be described by

5 $$\begin{aligned}& \tilde{Z}_{x}(k)=\alpha_{k}Z_{x}(k)+(1-\alpha _{k})Z_{x}(k-1), \\& \tilde{Z}_{y}(k)=\beta_{k}Z_{y}(k)+(1- \beta_{k})Z_{y}(k-1), \end{aligned}$$

where the stochastic variables $\alpha_{k},\beta_{k}\in\mathbb{R}$ are Bernoulli allocated with sequences directed by

6 $$\begin{aligned}& \operatorname{Prob}\{\alpha_{k}=1\}=\mathbb{E}\{\alpha_{k} \} =\alpha_{0},\qquad \operatorname{Prob}\{\alpha_{k}=0\}=1- \mathbb{E}\{\alpha_{k}\} =1-\alpha_{0}, \\& \operatorname{Prob}\{\beta_{k}=1\}=\mathbb{E}\{\beta_{k} \}=\beta_{0}, \qquad\operatorname{Prob}\{\beta_{k}=0\}=1-\mathbb{E} \{\beta_{k}\} =1-\beta_{0}. \end{aligned}$$

Here $\alpha_{0},\beta_{0}>0$ are known constants. Obviously, for $\alpha_{k}$, $\beta_{k}$, the variance $\sigma_{\alpha}=\alpha _{0}(1-\alpha_{0})$, $\sigma_{\beta}=\beta_{0}(1-\beta_{0})$.

The GRN state estimator to be designed is given as follows:

7 $$ \textstyle\begin{cases} \hat{x}(k+1)=-\mathbb{A}_{x}\hat{x}(k)+\mathbb{B}_{x}\tilde {Z}_{x}(k),\\ \hat{y}(k+1)=-\mathbb{A}_{y}\hat{y}(k)+\mathbb{B}_{y}\tilde {Z}_{y}(k), \end{cases} $$

where $\hat{x}(k),\hat{y}(k)\in\mathbb{R}^{n}$ are the estimations of $x(k)$ and $y(k)$, and $\mathbb{A}_{x}$, $\mathbb{A}_{y}$, $\mathbb {B}_{x}$, $\mathbb{B}_{y}$ are the estimator gain matrices to be determined.

Assume that the estimation error vectors are $\tilde{x}(k)=x(k)-\hat {x}(k)$ and $\tilde{y}(k)=y(k)-\hat{y}(k)$; the estimation error dynamics can be defined as follows from equations (1), (5), and (7):

8 $$\begin{aligned}& \begin{aligned} \tilde{x}(k+1)={}&{-}\bigl(\mathbb{A}+\Delta\mathbb{A}(k)\bigr)x(k-\rho _{1})+(\mathbb{A}_{x}-\alpha_{k} \mathbb{B}_{x}M)x(k)+\bigl(\mathbb {B}+\Delta\mathbb{B}(k)\bigr) \hat{g}\bigl(y\bigl(k-\delta(k)\bigr)\bigr) \\ &+\bigl(E+\Delta E(k)\bigr)\sum_{s=1}^{\infty} \mu_{s}h\bigl(y(k-s)\bigr)+\sigma \bigl(k,x(k-\rho_{1}) \bigr)\omega(k)-\mathbb{A}_{x}\tilde{x}(k) \\ &-(1-\alpha_{k})\mathbb{B}_{x}M x(k-1)+L_{x}v_{x}(k), \end{aligned} \\& \begin{aligned}[b] \tilde{y}(k+1)={}&{-}\bigl(\mathbb{C}+\Delta\mathbb{C}(k)\bigr)y(k-\rho _{2})+(\mathbb{A}_{y}-\beta_{k} \mathbb{B}_{y}N)y(k)+\bigl(\mathbb {D}+\Delta\mathbb{D}(k)\bigr) x \bigl(k-\tau(k)\bigr) \\ &+\bigl(F+\Delta F(k)\bigr)\sum_{n=1}^{\infty} \xi_{n}x\bigl((k-n)\bigr)-\mathbb {A}_{y}\tilde{y}(k)-(1- \beta_{k})\mathbb{B}_{y}N y(k-1) \\ &+L_{y}v_{y}(k). \end{aligned} \end{aligned}$$

For suitability, we denote

$$\begin{gathered} \bar{x}(k)= \left [ \textstyle\begin{array}{c} x(k)\\ \tilde{x}(k) \end{array}\displaystyle \right ] ,\qquad \bar{y}(k)= \left [ \textstyle\begin{array}{c} y(k)\\ \tilde{y}(k) \end{array}\displaystyle \right ], \\\bar{x}(j)=\psi(j),\quad j=-\tau_{M},-\tau_{M+1},\ldots,-1,0, \\ \bar{y}(j)=\varphi(j),\quad j=-\delta_{M},-\delta_{M+1},\ldots,-1,0,\end{gathered} $$

where $\psi(j)$, $j=-\tau_{M},-\tau_{M}+1,\ldots,-1,0$ and $\varphi(j)$, $j=-\delta_{M},-\delta_{M}+1,\ldots,-1,0$ are the initial conditions.

## Preliminaries

*Notations*: Throughout the paper, $\mathit{naturals}^{+}$ refers to the position for the set of nonnegative integers; $\mathbb{R}^{n}$ indicates the *n*-dimensional Euclidean space. The superscript “*T*” acts as the matrix transposition. The code $X\geq Y$ (each $X>Y$), where *X* and *Y* are symmetric matrices, means that $X-Y$ is positive semi-definitive (respectively positive definite). *I* means the identity matrix with consistent dimension. The symbol “∗” denotes the term symmetry. In addition, $E\{\cdot\}$ denotes the expectation operator. $L_{2}[0,\infty)$ is the amplitude of square-integrable vector functions over $[0,\infty)$. $|\cdot|$ denotes the Euclidean vector norm. Matrices, if not absolutely specified, are affected to have compatible dimensions.

### Assumption 1

The parameter uncertainties $\Delta\mathbb{A}(k)$, $\Delta\mathbb {B}(k)$, $\Delta\mathbb{C}(k)$, $\Delta\mathbb{D}(k)$, $\Delta E(k)$, $\Delta F(k)$ are of the following form.

The admissible parameter uncertainties are assumed to be of the form:

$$\begin{gathered} \bigl[\textstyle\begin{array}{c@{\quad}c@{\quad}c@{\quad}c@{\quad}c@{\quad}c}\Delta\mathbb{A}(k) &\Delta\mathbb{B}(k)& \Delta\mathbb{C}(k)& \Delta \mathbb{D}(k)& \Delta E(k) &\Delta F(k)\end{array}\displaystyle \bigr]\\\quad=R N(k) [\textstyle\begin{array}{@{}c@{\quad}c@{\quad}c@{\quad}c@{\quad}c@{\quad}c}W_{1}& W_{2}& W_{3}& W_{4} &W_{5}& W_{6}\end{array}\displaystyle ],\end{gathered} $$

where *R*, $\mathbb{W}_{i}$ ($i=1,2,\ldots,6$) are the known constant matrices with appropriate dimensions. The uncertain matrix $N(k)$ satisfies $N^{T}(k)N(k)\leq I$, $\forall k\in \mathit{naturals}^{+}$.

### Assumption 2

The vector-valued function $\hat{g}_{i}(\cdot)$ is assumed to satisfy the following sector-bounded condition, namely for $\forall x, y\in \mathbb{R}^{n}$:

$$ \bigl[\hat{g}(x)-\hat{g}(y)-N_{1}(x-y)\bigr]^{T} \bigl[ \hat{g}(x)-\hat {g}(y)-N_{2}(x-y)\bigr]\leq0, $$

where $N_{1}$, $N_{2}$ are known real constant matrices, and $\tilde {N}=N_{1}-N_{2}$ is a symmetric positive definite matrix.

### Definition 2.1

If there exist constants $\alpha>0$ and $0<\mu<1$, system (8) with $v_{x}(k)=0$ and $v_{y}(k)=0$ is global robust exponential state estimator of GRNs (1) with measurements (5) in the mean square sense such that

$$ \mathbb{E}\bigl\{ \big|\bar{x}(k)\big|^{2}+\big|\bar{y}(k)\big|^{2}\bigr\} \leq\alpha\mu ^{k}\Bigl(\max_{-\tau_{M}\leq k\leq0}\big| \bar{x}(k)\big|^{2}+\max_{-\delta _{M}\leq k\leq0}\big|\bar{y}(k)\big|^{2} \Bigr). $$

### Definition 2.2

If there exists a scalar $\gamma>0$, system (8) is a robust $H_{\infty}$ state estimator of GRNs (1) with measurements (5) in the mean square sense with zero initial conditions such that

$$ \mathbb{E}\sum_{k=0}^{\infty}\bigl\{ \big| \bar{x}(k)\big|^{2}+\big|\bar{y}(k)\big|^{2}\bigr\} \leq \gamma^{2} \mathbb{E}\sum_{k=0}^{\infty} \bigl(\big|v_{x}(k)\big|^{2}+\big|v_{y}(k)\big|^{2} \bigr) $$

for all non-zero $v_{x}(k),v_{y}(k)\in L_{2}[0,\infty)$.

The following lemmas are crucial in implementing our main results.

### Lemma 2.3

(see [2, 26])

*Let* *N* *and* *S* *be real constant matrices*; *matrix* $F(k)$ *satisfies* $F^{T}(k)F(k)\leq1$. *Then we have*:

(i) *For any* $\epsilon>0$, $NF(k)S+S^{T}F^{T}(k)N^{T}\leq \epsilon^{-1}NN^{T}+\epsilon S^{T}S$.
(ii) *For any* $P>0$, $\pm2 x^{T}y\leq x^{T}P^{-1}x+y^{T}Py$.

### Lemma 2.4

*Given the constant matrices* $\hat{\Omega}_{1}$, $\hat{\Omega}_{2}$, *and* $\hat{\Omega}_{3}$, *where* $\hat{\Omega}_{1}^{T}=\hat{\Omega }_{1}$ *and* $\hat{\Omega}_{2}^{T}=\hat{\Omega}_{2}>0$, *then* $\hat {\Omega}_{1}+\hat{\Omega}_{3}^{T}\hat{\Omega}_{2}^{-1}\hat{\Omega }_{3}<0$, *if and only if*

$$ \left [ \textstyle\begin{array}{c@{\quad}c} \hat{\Omega}_{1}& \hat{\Omega}_{3}^{T}\\ \hat{\Omega}_{3}& -\hat{\Omega}_{2} \end{array}\displaystyle \right ] < 0 \quad\textit{or} \quad \left [ \textstyle\begin{array}{c@{\quad}c} -\hat{\Omega}_{2}& \hat{\Omega}_{3}\\ \hat{\Omega}_{3}^{T}& \hat{\Omega}_{1} \end{array}\displaystyle \right ] < 0. $$

### Lemma 2.5

*Let* $\mathbb{M}\in\mathbb{R}^{n\times n}$ *be a positive semi*-*definite matrix*, $x_{i}\in\mathbb{R}^{n}$, *and* $a_{i}\geq0$ ($i=1,2,\ldots$). *If the series distressed are convergent*, *the following inequality holds*:

$$ \Biggl(\sum_{i=1}^{+\infty} a_{i}x_{i} \Biggr)^{T}\mathbb{M}\Biggl(\sum_{i=1}^{+\infty } a_{i}x_{i}\Biggr)\leq\Biggl(\sum _{i=1}^{+\infty} a_{i}\Biggr)\sum _{i=1}^{+\infty} a_{i}x_{i}^{T} \mathbb{M} x_{i}. $$

### Remark 2.1

In [1] Wang et al. investigated the robust state estimation for stochastic genetic regulatory networks with probabilistic delays in discrete sense, and Lv et al. [4] developed the robust distributed state estimation for genetic regulatory networks with Markovian jumping parameters. However, the inclusion of discrete-interval GRNs with leakage delays, probabilistic measurement delays, noise, and distributed delays has not been taken into account. So, the prime intention of this work is to elucidate that the state estimation problem for the improved system (8) with leakage delays is robustly exponentially stable.

## Exponential stability criterion

In this part, we first introduce a sufficient condition under which the augmented system (8) is robustly mean-square exponentially stable with the exogenous disturbance signals $v_{x}(k)=0$ and $v_{y}(k)=0$.

### Theorem 3.1

*Suppose that Assumptions* 1 *and* 2 *hold*. *Let the leakage delays* $\rho_{1}$, $\rho_{2}$ *and the estimation parameters* $\mathbb{A}_{x}$, $\mathbb{B}_{x}$, $\mathbb{A}_{y}$, *and* $\mathbb {B}_{y}$ *be given and also the acceptable conditions hold*. *Then the estimation error system* (8) *with* $v_{x}(k)=0$ *and* $v_{y}(k)=0$ *is robustly exponentially stable in the mean square if there exist positive definite matrices* $R_{11}$, $R_{12}$, $R_{21}$, $R_{22}$, $R_{31}$, $R_{32}$, $R_{41}$, $R_{42}$, $R_{51}$, $R_{52}$ *and three positive constant scalars* *λ*, $\varepsilon_{1}$, *and* $\varepsilon_{2}$ *such that the following LMI holds*:

9 $$ \Lambda_{1}= \left [ \textstyle\begin{array}{c@{\quad}c @{\quad}c} \Lambda'_{11}& \ast& \ast\\ S_{1}& J_{1}& \ast\\ 0& \bar{T}_{1}^{T}& -\varepsilon_{1}I \end{array}\displaystyle \right ]< 0,\qquad \Lambda_{2}= \left [ \textstyle\begin{array}{c@{\quad} c@{\quad} c} \Lambda'_{22}& \ast& \ast\\ S_{2}& J_{2}& \ast\\ 0& \bar{T}_{2}^{T}& -\varepsilon_{2}I \end{array}\displaystyle \right ]< 0, $$

*where*

$$ \begin{gathered} \Lambda'_{11}= \left [ \textstyle\begin{array}{c@{\quad}c@{\quad}c@{\quad}c@{\quad}c@{\quad }c@{\quad}c} \psi_{11}& 0& 0& 0& 0& 0& 0 \\ 0& -R_{21}& 0& 0& 0& 0& 0\\ 0& 0& -R_{31}& 0& 0& 0& 0\\ 0& 0& 0& -R_{41}+\varepsilon_{1}W_{4}^{T}W_{4}& 0& 0& 0\\ 0& 0& 0& 0& HR_{11}& 0& 0\\ 0& 0& 0& 0& 0& I(R_{12}+R_{22})& 0\\ 0& 0& 0& 0& 0& 0& -\bar{\xi}R_{52} \end{array}\displaystyle \right ], \\\Lambda'_{22}= \left [ \textstyle\begin{array}{c@{\quad}c@{\quad}c@{\quad}c@{\quad}c@{\quad }c@{\quad}c@{\quad}c@{\quad}c} \psi_{12}& 0& 0& 0& 0& 0& 0& 0& 0 \\ 0& -R_{22}& 0& 0& 0& 0& 0& 0& 0\\ 0& 0& -R_{32}& 0& 0& 0& 0& 0& 0\\ 0& 0& 0& -R_{42}-\lambda\tilde{N}_{1}+\varepsilon_{2}W_{2}^{T}W_{2}& -\lambda\tilde{N}_{2}^{T}& 0& 0& 0& 0\\ 0& 0& 0& -\lambda\tilde{N}_{2}& -\lambda I& 0& 0& 0& 0\\ 0& 0& 0& 0& 0& 0& 0& 0& 0\\ 0& 0& 0& 0& 0& 0& I(R_{11}+R_{21})& 0& 0\\ 0& 0& 0& 0& 0& 0& 0& \bar{\mu}R_{51}& 0\\ 0& 0& 0& 0& 0& 0& 0& 0& -\bar{\mu}R_{51} \end{array}\displaystyle \right ], \\S_{1}= \left [ \textstyle\begin{array}{c@{\quad}c@{\quad}c@{\quad}c@{\quad}c@{\quad }c@{\quad}c} 0& 0& 0& 0& \bar{\Xi}_{15}& 0& 0\\ \Xi_{21}& -\sqrt{2}R_{21}\mathbb{A}_{x}& \Xi_{23}& 0& 0& 0& 0\\ \sqrt{\sigma_{\alpha}}R_{21}\mathbb{B}_{x}M& 0& \sqrt{\sigma _{\alpha}}R_{21}\mathbb{B}_{x}M& 0& 0& 0& 0\\ 0& 0& 0& \bar{\Xi}_{44}& 0& 0& 0\\ 0& 0& 0& 0& 0& \bar{\Xi}_{55}& 0 \end{array}\displaystyle \right ],\end{gathered} $$

*where*

$$\begin{gathered} \bar{\Xi}_{15}=-\sqrt{2}(R_{11}+R_{21}) \mathbb{A};\quad\quad \bar{\Xi }_{44}=\sqrt{2}(R_{12}+R_{22}) \mathbb{D};\quad\quad \bar{\Xi}_{55}=\sqrt {2}(R_{12}+R_{22})F; \\ \Xi_{21}=\sqrt{2}R_{21}(\mathbb{A}_{x}- \alpha_{0}\mathbb {B}_{x}M); \qquad\Xi_{23}=- \sqrt{2}R_{21}(1-\alpha_{0})\mathbb{B}_{x}M; \\S_{2}= \left [ \textstyle\begin{array}{c@{\quad} c@{\quad} c@{\quad} c@{\quad} c@{\quad} c@{\quad} c@{\quad} c@{\quad} c} 0& 0& 0& 0& 0& \bar{\Theta}_{16}& 0& 0& 0\\ \Theta_{21}& -\sqrt{2}R_{22}\mathbb{A}_{y}& \Theta_{23}& 0& 0& 0& 0& 0& 0\\ \sqrt{\sigma_{\beta}}R_{22}\mathbb{B}_{y}N& 0& \sqrt{\sigma _{\beta}}R_{22}\mathbb{B}_{y}N& 0& 0& 0& 0& 0& 0\\ 0& 0& 0& 0& \bar{\Theta}_{45}& 0& 0& 0& 0\\ 0& 0& 0& 0& 0& 0& \bar{\Theta}_{57}& 0& 0 \end{array}\displaystyle \right ],\end{gathered} $$

*where*

$$\begin{aligned}& \bar{\Theta}_{16}=-\sqrt{2}(R_{12}+R_{22}) \mathbb{C}; \qquad\bar {\Theta}_{45}=\sqrt{2}(R_{11}+R_{21}) \mathbb{B}; \qquad\bar{\Theta }_{57}=\sqrt{2}(R_{11}+R_{21})E; \\& \Theta_{21}=\sqrt{2}R_{22}(\mathbb{A}_{y}- \beta_{0}\mathbb {B}_{y}N);\qquad \Theta_{23}=- \sqrt{2}R_{22}(1-\beta_{0})\mathbb {B}_{y}N, \\& J_{1}=\operatorname{diag}\bigl\{ -(R_{11}+R_{21}),-R_{21},-R_{21},-(R_{12}+R_{22}),-(R_{12}+R_{22}) \bigr\} , \\& J_{2}=\operatorname{diag}\bigl\{ -(R_{12}+R_{22}),-R_{22},-R_{22},-(R_{11}+R_{21}),-(R_{11}+R_{21}) \bigr\} , \\& \bar{T_{1}}= \left [ \textstyle\begin{array}{c@{\quad}c@{\quad}c@{\quad}c@{\quad}c@{\quad }c@{\quad}c} 0& 0& 0& 0& -\sqrt{2}(R_{11}+R_{21})T& 0& 0\\ 0& 0& 0& 0& 0& 0& 0\\ 0& 0& 0& 0& 0& 0& 0\\ 0& 0& 0& \sqrt{2}(R_{12}+R_{22})T& 0& 0& 0\\ 0& 0& 0& 0& 0& \sqrt{2}(R_{12}+R_{22})T& 0 \end{array}\displaystyle \right ], \\& \bar{T_{2}}= \left [ \textstyle\begin{array}{c@{\quad}c@{\quad}c@{\quad}c@{\quad}c@{\quad }c@{\quad}c@{\quad}c@{\quad}c} 0& 0& 0& 0& 0& -\sqrt{2}(R_{12}+R_{22})T& 0& 0& 0\\ 0& 0& 0& 0& 0& 0& 0& 0& 0\\ 0& 0& 0& 0& 0& 0& 0& 0& 0\\ 0& 0& 0& 0& \sqrt{2}(R_{11}+R_{21})T& 0& 0& 0& 0\\ 0& 0& 0& 0& 0& 0& \sqrt{2}(R_{11}+R_{21})T& 0& 0 \end{array}\displaystyle \right ], \\& \bar{\mu}=\sum_{s=1}^{\infty}\mu_{s},\qquad \bar{\xi}=\sum_{n=1}^{\infty}\xi_{n}, \\& \psi_{11}=-R_{11}+R_{31}+(\tau_{M}- \tau_{m}+1)R_{41}+\bar{\xi}R_{52};\qquad \psi_{12}=-R_{12}+R_{32}+(\delta_{M}- \delta_{m}+1)R_{42}, \\& \tilde{N}_{1}=\frac{(N_{1}^{T}N_{2}+N_{2}^{T}N_{1})}{2};\qquad \tilde {N}_{2}=- \frac{(N_{1}^{T}+N_{2}^{T})}{2}. \end{aligned}$$

### Proof

Choose a Lyapunov–Krasovskii functional for the augmented system (8):

10 $$ \mathbb{V}(k)=\mathbb{V}_{1}(k)+\mathbb{V}_{2}(k)+\mathbb {V}_{3}(k)+\mathbb{V}_{4}(k)+\mathbb{V}_{5}(k)+ \mathbb{V}_{6}(k), $$

where

$$\begin{gathered} \mathbb{V}_{1}(k)=x^{T}(k)R_{11}x(k)+y^{T}(k)R_{12}y(k), \\ \mathbb{V}_{2}(k)=\tilde{x}^{T}(k)R_{21} \tilde{x}(k)+\tilde {y}^{T}(k)R_{22}\tilde{y}(k), \\ \mathbb{V}_{3}(k)=x^{T}(k-1)R_{31}x(k-1)+y^{T}(k-1)R_{32}y(k-1), \\ \mathbb{V}_{4}(k)=\sum_{i=k-\tau(k)}^{k-1}x^{T}(i)R_{41}x(i)+ \sum_{i=k-\delta(k)}^{k-1}y^{T}(i)R_{42}y(i), \\ \mathbb{V}_{5}(k)=\sum_{j=-\tau_{M}+1}^{-\tau_{m}} \sum_{i=k+j}^{k-1}x^{T}(i)R_{41}x(i) +\sum_{j=-\delta_{M}+1}^{-\delta_{m}}\sum _{i=k+j}^{k-1}y^{T}(i)R_{42}y(i), \\ \mathbb{V}_{6}(k)=\sum_{i=1}^{\infty} \mu_{i}\sum_{j=k-i}^{k-1}h^{T} \bigl(y(j)\bigr) R_{51}h\bigl(y(j)\bigr)+\sum _{i=1}^{\infty}\xi_{i}\sum _{j=k-i}^{k-1}x^{T}(i)R_{52}x(i).\end{gathered} $$

Calculate the difference of $\mathbb{V}_{i}(k)$ ($i=1,2,\ldots,6$) along the trajectories of model (8) with $v_{x}(k)=0$, $v_{y}(k)=0$ and

11 $$ \mathbb{E}\bigl\{ \Delta\mathbb{V}(k)\bigr\} =\sum_{i=1}^{6} \mathbb{E}\bigl\{ \mathbb{V}_{i}(k)\bigr\} . $$

Now, we have

12 $$\begin{aligned}& \mathbb{E}\bigl\{ \Delta\mathbb{V}_{1}(k)\bigr\} =\mathbb{E}\bigl\{ \mathbb {V}_{1}(k+1)-\mathbb{V}_{1}(k)\bigr\} \\& \phantom{\mathbb{E}\bigl\{ \Delta\mathbb{V}_{1}(k)\bigr\} }=\mathbb{E}\Biggl\{ \Biggl[-\bigl(\mathbb{A}+\Delta\mathbb{A}(k)\bigr)x(k-\rho _{1})+\bigl(\mathbb{B}+\Delta\mathbb{B}(k)\bigr) \hat{g}\bigl(y \bigl(k-\delta (k)\bigr)\bigr) \\& \phantom{\mathbb{E}\bigl\{ \Delta\mathbb{V}_{1}(k)\bigr\} =}+\bigl(E+\Delta E(k)\bigr)\sum_{s=1}^{\infty}\mu _{s}h\bigl(y(k-s)\bigr)\Biggr]^{T} \\& \phantom{\mathbb{E}\bigl\{ \Delta\mathbb{V}_{1}(k)\bigr\} =}\times R_{11}\Biggl[- \bigl(\mathbb{A}+\Delta\mathbb{A}(k)\bigr)x(k-\rho _{1})+\bigl( \mathbb{B}+\Delta\mathbb{B}(k)\bigr) \hat{g}(y\bigl(k-\delta (k)\bigr) \\& \phantom{\mathbb{E}\bigl\{ \Delta\mathbb{V}_{1}(k)\bigr\} =}+\bigl(E+\Delta E(k)\bigr)\sum_{s=1}^{\infty} \mu_{s}h\bigl(y(k-s)\bigr)\Biggr] \\& \phantom{\mathbb{E}\bigl\{ \Delta\mathbb{V}_{1}(k)\bigr\} =}+\sigma ^{T}\bigl(k,x(k- \rho_{1})\bigr)R_{11}\sigma\bigl(k,x(k-\rho_{1}) \bigr)-x^{T}(k)R_{11}x(k)-y^{T}(k)R_{12}y(k) \\& \phantom{\mathbb{E}\bigl\{ \Delta\mathbb{V}_{1}(k)\bigr\} =}+ \Biggl[-\bigl(\mathbb{C}+\Delta \mathbb{C}(k)\bigr)y(k-\rho_{2}) +\bigl( \mathbb{D}+\Delta\mathbb{D}(k)\bigr)x\bigl(k-\tau(k)\bigr) \\& \phantom{\mathbb{E}\bigl\{ \Delta\mathbb{V}_{1}(k)\bigr\} =}+\bigl(F+\Delta F(k)\bigr)\sum _{n=1}^{\infty}\xi _{n}x(k-n) \Biggr]^{T} \\& \phantom{\mathbb{E}\bigl\{ \Delta\mathbb{V}_{1}(k)\bigr\} =}\times R_{12}\Biggl[-\bigl(\mathbb{C}+\Delta\mathbb{C}(k) \bigr)y(k-\rho _{2})+\bigl(\mathbb{D}+\Delta\mathbb{D}(k)\bigr)x\bigl(k-\tau(k)\bigr) \\& \phantom{\mathbb{E}\bigl\{ \Delta\mathbb{V}_{1}(k)\bigr\} =}+ \bigl(F+\Delta F(k)\bigr)\sum_{n=1}^{\infty} \xi_{n}x(k-n)\Biggr]\Biggr\} , \end{aligned}$$

13 $$\begin{aligned}& \mathbb{E}\bigl\{ \Delta\mathbb{V}_{2}(k)\bigr\} =\mathbb{E}\bigl\{ \mathbb {V}_{2}(k+1)-\mathbb{V}_{2}(k)\bigr\} \\& \phantom{\mathbb{E}\bigl\{ \Delta\mathbb{V}_{2}(k)\bigr\} }=\mathbb{E}\Biggl\{ \Biggl[-\bigl(\mathbb{A}+\Delta\mathbb{A}(k)\bigr)x(k-\rho _{1})+(\mathbb{A}_{x}-\alpha_{k} \mathbb{B}_{x}M)x(k) \\& \phantom{\mathbb{E}\bigl\{ \Delta\mathbb{V}_{2}(k)\bigr\} =}+\bigl(\mathbb {B}+\Delta\mathbb{B}(k)\bigr) \hat{g}\bigl(y\bigl(k-\delta(k)\bigr)\bigr)+\bigl(E+\Delta E(k)\bigr)\sum_{s=1}^{\infty} \mu_{s}h\bigl(y(k-s)\bigr) \\& \phantom{\mathbb{E}\bigl\{ \Delta\mathbb{V}_{2}(k)\bigr\} =}-\mathbb {A}_{x}\tilde{x}(k)-(1- \alpha_{k})\mathbb{B}_{x}M x(k-1)\Biggr]^{T} \\& \phantom{\mathbb{E}\bigl\{ \Delta\mathbb{V}_{2}(k)\bigr\} =}\times R_{21}\Biggl[-\bigl(\mathbb{A}+\Delta\mathbb{A}(k)\bigr)x(k- \rho_{1})+(\mathbb {A}_{x}-\alpha_{k} \mathbb{B}_{x}M)x(k) \\& \phantom{\mathbb{E}\bigl\{ \Delta\mathbb{V}_{2}(k)\bigr\} =}+\bigl(\mathbb{B}+\Delta\mathbb {B}(k)\bigr) \hat{g}\bigl(y\bigl(k-\delta(k)\bigr)\bigr)+\bigl(E+\Delta E(k)\bigr)\sum_{s=1}^{\infty} \mu_{s}h\bigl(y(k-s)\bigr) \\& \phantom{\mathbb{E}\bigl\{ \Delta\mathbb{V}_{2}(k)\bigr\} =}-\mathbb {A}_{x}\tilde{x}(k)-(1- \alpha_{k})\mathbb{B}_{x}M x(k-1)\Biggr] \\& \phantom{\mathbb{E}\bigl\{ \Delta\mathbb{V}_{2}(k)\bigr\} =}+\sigma_{\alpha}\bigl[\mathbb{B}_{x}M x(k)+ \mathbb{B}_{x} M x(k-1)\bigr]^{T}R_{21}\bigl[ \mathbb{B}_{x}M x(k)+\mathbb{B}_{x} M x(k-1)\bigr] \\& \phantom{\mathbb{E}\bigl\{ \Delta\mathbb{V}_{2}(k)\bigr\} =}- \tilde{x}^{T}(k)R_{21}\tilde{x}(k)+\Biggl[-\bigl(\mathbb{C}+\Delta\mathbb{C}(k)\bigr)y(k- \rho_{2})+(\mathbb {A}_{y}-\beta_{k} \mathbb{B}_{y}N)y(k) \\& \phantom{\mathbb{E}\bigl\{ \Delta\mathbb{V}_{2}(k)\bigr\} =}+\bigl(\mathbb{D}+\Delta\mathbb {D}(k)\bigr) x \bigl(k-\tau(k)\bigr)+\bigl(F+\Delta F(k)\bigr)\sum_{n=1}^{\infty} \xi_{n}x\bigl((k-n)\bigr) \\& \phantom{\mathbb{E}\bigl\{ \Delta\mathbb{V}_{2}(k)\bigr\} =}-\mathbb {A}_{y}\tilde{y}(k)-(1- \beta_{k})\mathbb{B}_{y}N y(k-1)\Biggr]^{T} \\& \phantom{\mathbb{E}\bigl\{ \Delta\mathbb{V}_{2}(k)\bigr\} =}\times R_{22}\Biggl[-\bigl(\mathbb{C}+\Delta\mathbb{C}(k)\bigr)y(k- \rho_{2})+(\mathbb {A}_{y}-\beta_{k} \mathbb{B}_{y}N)y(k) \\& \phantom{\mathbb{E}\bigl\{ \Delta\mathbb{V}_{2}(k)\bigr\} =}+\bigl(\mathbb{D}+\Delta\mathbb {D}(k)\bigr) x \bigl(k-\tau(k)\bigr)+\bigl(F+\Delta F(k)\bigr)\sum_{n=1}^{\infty} \xi_{n}x\bigl((k-n)\bigr) \\& \phantom{\mathbb{E}\bigl\{ \Delta\mathbb{V}_{2}(k)\bigr\} =}-\mathbb {A}_{y}\tilde{y}(k)-(1- \beta_{k})\mathbb{B}_{y}N y(k-1)\Biggr] \\& \phantom{\mathbb{E}\bigl\{ \Delta\mathbb{V}_{2}(k)\bigr\} =}+\sigma_{\beta}\bigl[\mathbb{B}_{y}N y(k)+ \mathbb{B}_{y} N y(k-1)\bigr]^{T}R_{22}\bigl[ \mathbb{B}_{y}N y(k)+\mathbb{B}_{y} N y(k-1)\bigr] \\& \phantom{\mathbb{E}\bigl\{ \Delta\mathbb{V}_{2}(k)\bigr\} =}-\tilde{y}^{T}(k)R_{22}\tilde{y}(k)\Biggr\} , \end{aligned}$$

14 $$\begin{aligned}& \begin{aligned}[b] \mathbb{E}\bigl\{ \Delta\mathbb{V}_{3}(k)\bigr\} ={}&\mathbb{E}\bigl\{ \mathbb {V}_{3}(k+1)-\mathbb{V}_{3}(k)\bigr\} \\ ={}&\mathbb{E}\bigl\{ x^{T}(k)R_{31}x(k)-x^{T}(k-1)R_{31}x(k-1) \\ &+y^{T}(k)R_{32}y(k)-y^{T}(k-1)R_{32}y(k-1)\bigr\} , \end{aligned} \end{aligned}$$

15 $$\begin{aligned}& \begin{aligned}[b] \mathbb{E}\bigl\{ \Delta\mathbb{V}_{4}(k)\bigr\} ={}&\mathbb{E}\bigl\{ \mathbb {V}_{4}(k+1)-\mathbb{V}_{4}(k)\bigr\} \\ \leq{}& \mathbb{E}\Biggl\{ x^{T}(k)R_{41}x(k)-x^{T} \bigl(k-\tau(k)\bigr)R_{41}x\bigl(k-\tau (k)\bigr)\\ &+\sum _{i=k-\tau_{M}+1}^{k-\tau_{m}}x^{T}(i)R_{41}x(i) \\ &+y^{T}(k)R_{42}y(k)-y^{T}\bigl(k-\delta(k) \bigr)R_{42}y\bigl(k-\delta (k)\bigr)\\ &+\sum_{i=k-\delta_{M}+1}^{k-\delta m}y^{T}(i)R_{42}y(i) \Biggr\} , \end{aligned} \end{aligned}$$

16 $$\begin{aligned}& \begin{aligned}[b] \mathbb{E}\bigl\{ \Delta\mathbb{V}_{5}(k)\bigr\} ={}&\mathbb{E}\bigl\{ \mathbb {V}_{5}(k+1)-\mathbb{V}_{5}(k)\bigr\} \\ ={}&\mathbb{E}\Biggl\{ (\tau_{M}-\tau_{m})x^{T}(k)R_{41}x(k)- \sum_{i=k-\tau _{M}+1}^{k-\tau_{m}}x^{T}(i)R_{41}x(i) \\ &+(\delta_{M}-\delta_{m})y^{T}(k)R_{42}y(k)- \sum_{i=k-\delta _{M}+1}^{k-\delta m}y^{T}(i)R_{42}y(i) \Biggr\} , \end{aligned} \\& \begin{aligned} \mathbb{E}\bigl\{ \Delta\mathbb{V}_{6}(k)\bigr\} ={}&\mathbb{E}\bigl\{ \mathbb {V}_{6}(k+1)-\mathbb{V}_{6}(k)\bigr\} \\ ={}&\sum_{i=1}^{\infty}\mu_{i}\sum _{j=k+1-i}^{k+1-1}h^{T}\bigl(y(j) \bigr)R_{51}h\bigl(y(j)\bigr)+\sum_{i=1}^{\infty} \xi _{i}\sum_{j=k+1-i}^{k+1-1}x^{T}(i)R_{52}x(i) \\ &-\sum_{i=1}^{\infty}\mu_{i} \sum_{j=k-i}^{k-1}h^{T}\bigl(y(j) \bigr)R_{51}h\bigl(y(j)\bigr)-\sum_{i=1}^{\infty} \xi _{i}\sum_{j=k-i}^{k-1}x^{T}(i)R_{52}x(i) \\ ={}&\sum_{i=1}^{\infty}\mu _{i} \bigl[h^{T}\bigl(y(k)\bigr)R_{51}h\bigl(y(k) \bigr)-h^{T}\bigl(y(k-i)\bigr)R_{51}h\bigl(y(k-i)\bigr)\bigr] \\ &+\sum_{i=1}^{\infty}\xi _{i} \bigl[x^{T}(k)R_{52}x(k)-x^{T}(k-i)R_{52}x(k-i) \bigr]. \end{aligned} \end{aligned}$$

Using Lemma 2.5, we get

17 $$\begin{aligned}[b] \mathbb{E}\bigl\{ \Delta\mathbb{V}_{6}(k)\bigr\} \leq{}& \bar{\mu }h^{T}\bigl(y(k)\bigr)R_{51}h\bigl(y(k)\bigr)-\bar{\mu}\bigl[ \bar{\mu }h\bigl(y(k-s)\bigr)\bigr]^{T}R_{51}\bigl[\bar{\mu}h \bigl(y(k-s)\bigr)\bigr] \\ &+\bar{\xi}x^{T}(k)R_{52}x(k)-\bar{\xi}\bigl[\bar{\xi }x(k-n)\bigr]^{T}R_{52}\bigl[\bar{\xi}x(k-n)\bigr].\end{aligned} $$

Substituting equations (12)–(17) into equation (11) results in

18 $$\begin{aligned}[b] \mathbb{E}\bigl\{ \Delta\mathbb{V}(k)\bigr\} \leq{}&\mathbb{E}\bigl\{ \varpi _{0}^{T}(k)\bigl[\Lambda_{11}+ \sigma_{\alpha}\hat{W}_{01}^{T}R_{21} \hat{W}_{01} +2\hat{G}_{01}^{T}(k) (R_{11}+R_{21})\hat{G}_{01}(k) \\ &+2\hat{F}_{01}^{T}(k)R_{21} \hat{F}_{01}(k)+2\hat {G}_{11}^{T}(k) (R_{12}+R_{22})\hat{G}_{11}(k) \\ &+2\hat{S}_{01}^{T}(k) (R_{12}+R_{22}) \hat{S}_{01}(k)\bigr]\varpi_{0}(k)\\ & +\Gamma_{0}^{T}(k) \bigl[\Lambda_{12}+\sigma_{\beta}\hat {W}_{02}^{T}R_{22} \hat{W}_{02} +2\hat{G}_{02}^{T}(k) (R_{12}+R_{22}) \hat{G}_{02}(k) \\ &+2\hat {F}_{02}^{T}(k)R_{22} \hat{F}_{02}(k)+2\hat{G}_{12}^{T}(k) (R_{11}+R_{21}) \hat{G}_{12}(k)\\ &+2\hat {S}_{02}^{T}(k) (R_{11}+R_{21})\hat{S}_{02}(k)\bigr] \Gamma_{0}(k)\bigr\} ,\end{aligned} $$

where

$$\begin{gathered} \varpi_{0}(k)=\bigl[x^{T}(k),\tilde{x}^{T}(k),x^{T}(k-1),x^{T} \bigl(k-\tau (k)\bigr),x^{T}(k-\rho_{1}),x^{T}(k-n), \bigl[\bar{\xi}x(k-n)\bigr]^{T}\bigr], \\ \begin{aligned}\Gamma_{0}(k)={}&\bigl[y^{T}(k),\tilde{y}^{T}(k),y^{T}(k-1),y^{T} \bigl(k-\delta (k)\bigr),g^{T}\bigl(y\bigl(k-\delta(k)\bigr) \bigr),y^{T}(k-\rho_{2}),h^{T}\bigl(y(k-s)\bigr), \\ & h^{T}(k),\bigl[\bar{\mu}h\bigl(y(k-s)\bigr) \bigr]^{T}\bigr],\end{aligned}\end{gathered} $$

where

$$\begin{aligned}& \Lambda_{11}= \left [ \textstyle\begin{array}{c@{\quad}c@{\quad}c@{\quad}c@{\quad}c@{\quad }c@{\quad}c} \psi_{11}& 0& 0& 0& 0& 0& 0 \\ 0& -R_{21}& 0& 0& 0& 0& 0\\ 0& 0& -R_{31}& 0& 0& 0& 0\\ 0& 0& 0& -R_{41}& 0& 0& 0\\ 0& 0& 0& 0& HR_{11}& 0& 0\\ 0& 0& 0& 0& 0& I(R_{12}+R_{22})& 0\\ 0& 0& 0& 0& 0& 0& -\bar{\xi}R_{52} \end{array}\displaystyle \right ], \\& \Lambda_{12}= \left [ \textstyle\begin{array}{c@{\quad}c@{\quad}c@{\quad}c@{\quad}c@{\quad }c@{\quad}c@{\quad}c@{\quad}c} \psi_{12}& 0& 0& 0& 0& 0& 0& 0& 0 \\ 0& -R_{22}& 0& 0& 0& 0& 0& 0& 0\\ 0& 0& -R_{32}& 0& 0& 0& 0& 0& 0\\ 0& 0& 0& -R_{42}& 0& 0& 0& 0& 0\\ 0& 0& 0& 0& 0& 0& 0& 0& 0\\ 0& 0& 0& 0& 0& 0& 0& 0& 0\\ 0& 0& 0& 0& 0& 0& I(R_{11}+R_{21})& 0& 0\\ 0& 0& 0& 0& 0& 0& 0& \bar{\mu}R_{51}& 0\\ 0& 0& 0& 0& 0& 0& 0& 0& -\bar{\mu}R_{51} \end{array}\displaystyle \right ], \\& \psi_{11}=-R_{11}+R_{31}+(\tau_{M}- \tau_{m}+1)R_{41}+\bar{\xi }R_{52};\qquad \psi_{12}=-R_{12}+R_{32}+(\delta_{M}- \delta_{m}+1)R_{42}, \\& \hat{W}_{01}=[\mathbb{B}_{x}M,0,\mathbb{B}_{x}M,0,0,0,0];\qquad \hat {W}_{02}=[\mathbb{B}_{y}N,0,\mathbb{B}_{y}N,0,0,0,0,0,0], \\& \hat{G}_{01}(k)=\bigl[0,0,0,0,-\bigl(\mathbb{A}+\Delta\mathbb{A}(k) \bigr),0,0\bigr]; \\& \hat{G}_{02}(k)=\bigl[0,0,0,0,0,-\bigl(\mathbb{C}+ \Delta\mathbb {C}(k)\bigr),0,0,0\bigr], \\& \hat{F}_{01}(k)=\bigl[\mathbb{A}_{x}- \alpha_{0}\mathbb {B}_{x}M,-\mathbb{A}_{x},-(1- \alpha_{0})\mathbb{B}_{x}M,0,0,0,0\bigr]; \\& \hat{F}_{02}(k)=\bigl[\mathbb{A}_{y}-\beta_{0} \mathbb{B}_{y}N,-\mathbb {A}_{y},-(1-\beta_{0}) \mathbb{B}_{y}N,0,0,0,0,0,0\bigr], \\& \hat{G}_{11}(k)=\bigl[0,0,0,\bigl(\mathbb{D}+\Delta\mathbb{D}(k) \bigr),0,0,0\bigr];\qquad \hat{G}_{12}(k)=\bigl[0,0,0,0,\bigl(\mathbb{B}+ \Delta\mathbb{B}(k)\bigr),0,0,0,0\bigr], \\& \hat{S}_{01}(k)=\bigl[0,0,0,0,0,\bigl(F+\Delta F(k)\bigr),0\bigr];\qquad \hat {S}_{02}(k)=\bigl[0,0,0,0,0,0,\bigl(E+\Delta E(k)\bigr),0,0\bigr]. \end{aligned}$$

From Assumption 2, we have

19 $$ \left [ \textstyle\begin{array}{c} y(k-\delta(k))\\ \hat{g}(y(k-\delta(k))) \end{array}\displaystyle \right ]^{T} \left [ \textstyle\begin{array}{c@{\quad}c} \tilde{N}_{1}& \tilde{N}_{2}\\ \tilde{N}_{2}^{T}& I \end{array}\displaystyle \right ] \left [ \textstyle\begin{array}{c} y(k-\delta(k))\\ \hat{g}(y(k-\delta(k))) \end{array}\displaystyle \right ]\leq0, $$

where

$$ \tilde{N}_{1}=\frac{(N_{1}^{T}N_{2}+N_{2}^{T}N_{1})}{2} ;\qquad \tilde {N}_{2}=- \frac{(N_{1}^{T}+N_{2}^{T})}{2}. $$

Then, from equations (18) and (19), we have

20 $$\begin{aligned}[b] \mathbb{E}\bigl\{ \Delta V(k)\bigr\} \leq{}&\mathbb{E}\bigl\{ \Delta V(k)\bigr\} - \mathbb {E}\left\{\lambda \left [ \textstyle\begin{array}{c} y(k-\delta(k))\\ g(y(k-\delta(k))) \end{array}\displaystyle \right ]^{T} \left [ \textstyle\begin{array}{c@{\quad}c} \tilde{N}_{1}& \tilde{N}_{2}\\ \tilde{N}_{2}^{T}& I \end{array}\displaystyle \right ] \left [ \textstyle\begin{array}{c} y(k-\delta(k))\\ g(y(k-\delta(k))) \end{array}\displaystyle \right ]\right\} \\ ={}&\mathbb{E}\bigl\{ \varpi_{0}^{T}(k)\bigl[ \Lambda_{11}+\sigma_{\alpha}\hat {W}_{01}^{T}R_{21} \hat{W}_{01} +2\hat{G}_{01}^{T}(k) (R_{11}+R_{21})\hat{G}_{01}(k) \\ &+2\hat{F}_{01}^{T}(k)R_{21} \hat{F}_{01}(k)+2\hat {G}_{11}^{T}(k) (R_{12}+R_{22})\hat{G}_{11}(k) \\ &+2\hat{S}_{01}^{T}(k) (R_{12}+R_{22}) \hat{S}_{01}(k)\bigr]\varpi_{0}(k)\\ & +\Gamma_{0}^{T}(k) \bigl[\Lambda_{22}+\sigma_{\beta}\hat {W}_{02}^{T}R_{22} \hat{W}_{02} +2\hat{G}_{02}^{T}(k) (R_{12}+R_{22}) \hat{G}_{02}(k) \\ &+2\hat {F}_{02}^{T}(k)R_{22} \hat{F}_{02}(k)+2\hat{G}_{12}^{T}(k) (R_{11}+R_{21}) \hat{G}_{12}(k)\\&+2\hat {S}_{02}^{T}(k) (R_{11}+P_{21})\hat{S}_{02}(k)\bigr] \Gamma_{0}(k)\bigr\} , \end{aligned} $$

where

$$ \Lambda_{22}= \left [ \textstyle\begin{array}{c@{\quad}c@{\quad}c@{\quad}c@{\quad}c@{\quad }c@{\quad}c@{\quad}c@{\quad}c} \psi_{12}& 0& 0& 0& 0& 0& 0& 0& 0 \\ 0& -R_{22}& 0& 0& 0& 0& 0& 0& 0\\ 0& 0& -R_{32}& 0& 0& 0& 0& 0& 0\\ 0& 0& 0& -R_{42}-\lambda\tilde{N}_{1}& -\lambda\tilde{N}_{2}^{T}& 0& 0& 0& 0\\ 0& 0& 0& -\lambda\tilde{N}_{2}& -\lambda I& 0& 0& 0& 0\\ 0& 0& 0& 0& 0& 0& 0& 0& 0\\ 0& 0& 0& 0& 0& 0& I(R_{11}+R_{21})& 0& 0\\ 0& 0& 0& 0& 0& 0& 0& \bar{\mu}R_{51}& 0\\ 0& 0& 0& 0& 0& 0& 0& 0& -\bar{\mu}R_{51} \end{array}\displaystyle \right ]. $$

Notice that, since $\Lambda_{1}<0$ and $\Lambda_{2}<0$, there are two scalars $\mu_{1}>0$ and $\mu_{2}>0$ such that

21 $$\begin{aligned}& \hat{\Lambda}_{1}=\Lambda_{1}+\mu_{1}\left [ \textstyle\begin{array}{c@{\quad}c} I_{2n\times2n}& 0\\ 0& 0 \end{array}\displaystyle \right ]< 0, \\& \hat{\Lambda}_{2}=\Lambda_{2}+\mu_{2}\left [ \textstyle\begin{array}{c@{\quad}c} I_{2n\times2n}& 0\\ 0& 0 \end{array}\displaystyle \right ]< 0. \end{aligned}$$

Equation (21) implies

22 $$\begin{aligned}& \Lambda_{11}+\mu_{1}\left [ \textstyle\begin{array}{c@{\quad}c} I_{2n\times2n}& 0 \\ 0& 0 \end{array}\displaystyle \right ]+\sigma_{\alpha}\hat{W}_{01}^{T}R_{21} \hat{W}_{01} +2\hat{G}_{01}^{T}(k) (R_{11}+R_{21})\hat{G}_{01}(k) +2 \hat{F}_{01}^{T}(k)R_{21}\hat{F}_{01}(k) \\& \quad+2\hat{G}_{11}^{T}(k) (R_{12}+R_{22}) \hat{G}_{11}(k) +2\hat{S}_{01}^{T}(k) (R_{12}+R_{22})\hat{S}_{01}(k)< 0, \\& \Lambda_{22}+\mu_{2}\left [ \textstyle\begin{array}{c@{\quad}c} I_{2n\times2n}& 0\\ 0& 0 \end{array}\displaystyle \right ]+\sigma_{\beta}\hat{W}_{02}^{T}R_{22} \hat{W}_{02} +2\hat{G}_{02}^{T}(k) (R_{12}+R_{22})\hat{G}_{02}(k)+2\hat {F}_{02}^{T}(k)R_{22}\hat{F}_{02}(k) \\& \quad+2\hat{G}_{12}^{T}(k) (R_{11}+R_{21}) \hat{G}_{12}(k)+2\hat {S}_{02}^{T}(k) (R_{11}+R_{21})\hat{S}_{02}(k)< 0. \end{aligned}$$

First we satisfy (21) before proving the exponential stability. Using Lemma 2.4, the above equalities are equivalent to

23 $$ \Lambda_{3}(k)= \left [ \textstyle\begin{array}{c@{\quad}c} \hat{\Lambda}_{11}& \ast\\ S_{1}(k)& J_{1} \end{array}\displaystyle \right ]< 0 ,\qquad \Lambda_{4}(k)= \left [ \textstyle\begin{array}{c@{\quad}c} \hat{\Lambda}_{22}& \ast\\ S_{2}(k)& J_{2} \end{array}\displaystyle \right ]< 0, $$

where

$$\begin{gathered} \hat{\Lambda}_{11}=\Lambda_{11}+\mu_{1}\left [ \textstyle\begin{array}{c@{\quad}c} I_{2n\times2n}& 0\\ 0& 0 \end{array}\displaystyle \right ], \\ \hat{\Lambda}_{22}=\Lambda_{22}+\mu_{2}\left [ \textstyle\begin{array}{c@{\quad}c} I_{2n\times2n}& 0\\ 0& 0 \end{array}\displaystyle \right ], \\\begin{aligned}S_{1}(k)&= \left [ \textstyle\begin{array}{c} \sqrt{2}(R_{11}+R_{21})\hat{G}_{01}(k)\\ \sqrt{2}R_{21}\hat{F}_{01}(k)\\ \sqrt{\sigma_{\alpha}}R_{21}\hat{W}_{01}\\ \sqrt{2}(R_{12}+R_{22})\hat{G}_{11}(k)\\ \sqrt{2}(R_{12}+R_{22})\hat{S}_{01}(k) \end{array}\displaystyle \right ] \\ &= \left [ \textstyle\begin{array}{c@{\quad}c@{\quad}c@{\quad}c@{\quad}c@{\quad }c@{\quad}c} 0& 0& 0& 0& \Xi_{15}& 0& 0\\ \Xi_{21}& -\sqrt{2}R_{21}\mathbb{A}_{x}& \Xi_{23}& 0& 0& 0& 0\\ \sqrt{\sigma_{\alpha}}R_{21}\mathbb{B}_{x}M& 0& \sqrt{\sigma _{\alpha}}R_{21}\mathbb{B}_{x}M& 0& 0& 0& 0\\ 0& 0& 0& \Xi_{44}& 0& 0& 0\\ 0& 0& 0& 0& 0& \Xi_{55}& 0 \end{array}\displaystyle \right ],\end{aligned}\end{gathered} $$

where

$$\begin{aligned}& \Xi_{15}=-\sqrt{2}(R_{11}+R_{21}) \bigl( \mathbb{A}+\Delta\mathbb {A}(k)\bigr) ;\\& \Xi_{21}=\sqrt{2}R_{21}( \mathbb{A}_{x}-\alpha_{0}\mathbb {B}_{x}M); \\& \Xi_{23}=-\sqrt{2}R_{21}(1-\alpha_{0}) \mathbb{B}_{x}M ;\\& \Xi_{44}=\sqrt{2}(R_{12}+R_{22}) \bigl(\mathbb{D}+\Delta\mathbb{D}(k)\bigr); \\& \Xi_{55}=\sqrt{2}(R_{12}+R_{22}) \bigl(F+\Delta F(k)\bigr), \\& \begin{aligned}S_{2}(k)&= \left [ \textstyle\begin{array}{c} \sqrt{2}(R_{12}+R_{22})\hat{G}_{02}(k)\\ \sqrt{2}R_{22}\hat{F}_{02}(k)\\ \sqrt{\sigma_{\beta}}R_{22}\hat{W}_{02}\\ \sqrt{2}(R_{11}+R_{21})\hat{G}_{12}(k)\\ \sqrt{2}(R_{11}+R_{21})\hat{S}_{02}(k) \end{array}\displaystyle \right ] \\ &= \left [ \textstyle\begin{array}{c@{\quad}c@{\quad}c@{\quad}c@{\quad}c@{\quad }c@{\quad}c@{\quad}c@{\quad}c} 0& 0& 0& 0& 0& \Theta_{16}& 0& 0& 0\\ \Theta_{21}& -\sqrt{2}R_{22}\mathbb{A}_{y}& \Theta_{23}& 0& 0& 0& 0& 0& 0\\ \sqrt{\sigma_{\beta}}R_{22}\mathbb{B}_{y}N& 0& \sqrt{\sigma _{\beta}}R_{22}\mathbb{B}_{y}N& 0& 0& 0& 0& 0& 0\\ 0& 0& 0& 0& \Theta_{45}& 0& 0& 0& 0\\ 0& 0& 0& 0& 0& 0& \Theta_{57}& 0& 0 \end{array}\displaystyle \right ],\end{aligned} \end{aligned}$$

where

$$\begin{gathered} \Theta_{16}=-\sqrt{2}(R_{12}+R_{22}) \bigl( \mathbb{C}+\Delta\mathbb {C}(k)\bigr);\qquad \Theta_{21}=\sqrt{2}R_{22}( \mathbb{A}_{y}-\beta_{0}\mathbb {B}_{y}N); \\ \Theta_{23}=-\sqrt{2}R_{22}(1-\beta_{0}) \mathbb{B}_{y}N;\qquad \Theta _{45}=\sqrt{2}(R_{11}+R_{21}) \bigl(\mathbb{B}+\Delta\mathbb{B}(k)\bigr); \\ \Theta_{57}=\sqrt{2}(R_{11}+R_{21}) \bigl(E+ \Delta E(k)\bigr), \\ J_{1}=\operatorname{diag}\bigl\{ -(R_{11}+R_{21}),-R_{21},-R_{21},-(R_{12}+R_{22}),-(R_{12}+R_{22}) \bigr\} , \\ J_{2}=\operatorname{diag}\bigl\{ -(R_{12}+R_{22}),-R_{22},-R_{22},-(R_{11}+R_{21}),-(R_{11}+R_{21}) \bigr\} .\end{gathered} $$

Note that $S_{1}(k)$ and $S_{2}(k)$ can be decomposed as

24 $$\begin{aligned}& S_{1}(k)=S_{1}+\Delta S_{1}(k), \\& S_{2}(k)=S_{2}+\Delta S_{2}(k), \end{aligned}$$

where

$$\begin{aligned}& S_{1}= \left [ \textstyle\begin{array}{c@{\quad}c@{\quad}c@{\quad}c@{\quad}c@{\quad }c@{\quad}c} 0& 0& 0& 0& \bar{\Xi}_{15}& 0& 0\\ \Xi_{21}& -\sqrt{2}R_{21}\mathbb{A}_{x}& \Xi_{23}& 0& 0& 0& 0\\ \sqrt{\sigma_{\alpha}}R_{21}\mathbb{B}_{x}M& 0& \sqrt{\sigma _{\alpha}}R_{21}\mathbb{B}_{x}M& 0& 0& 0& 0\\ 0& 0& 0& \bar{\Xi}_{44}& 0& 0& 0\\ 0& 0& 0& 0& 0& \bar{\Xi}_{55}& 0 \end{array}\displaystyle \right ], \\& \bar{\Xi}_{15}=-\sqrt{2}(R_{11}+R_{21}) \mathbb{A}; \quad\quad\bar{\Xi }_{44}=\sqrt{2}(R_{12} +R_{22}) \mathbb{D}; \\& \bar{\Xi}_{55}=\sqrt{2}(R_{12}+R_{22})F, \\& \Delta S_{1}(k)= \left [ \textstyle\begin{array}{c@{\quad}c@{\quad}c@{\quad}c@{\quad}c@{\quad }c@{\quad}c} 0& 0& 0& 0& -\sqrt{2}(R_{11}+R_{21})\Delta\mathbb{A}(k)& 0& 0\\ 0& 0& 0& 0& 0& 0& 0\\ 0& 0& 0& 0& 0& 0& 0\\ 0& 0& 0& \sqrt{2}(R_{12}+R_{22})\Delta\mathbb{D}(k)& 0& 0& 0\\ 0& 0& 0& 0& 0& \sqrt{2}(R_{12}+R_{22})\Delta\mathbb{F}(k)& 0 \end{array}\displaystyle \right ], \\& S_{2}= \left [ \textstyle\begin{array}{c@{\quad}c@{\quad}c@{\quad}c@{\quad}c@{\quad }c@{\quad}c@{\quad}c@{\quad}c} 0& 0& 0& 0& 0& \bar{\Theta}_{16}& 0& 0& 0\\ \Theta_{21}& -\sqrt{2}R_{22}\mathbb{A}_{y}& \Theta_{23}& 0& 0& 0& 0& 0& 0\\ \sqrt{\sigma_{\beta}}R_{22}\mathbb{B}_{y}N& 0& \sqrt{\sigma _{\beta}}R_{22}\mathbb{B}_{y}N& 0& 0& 0& 0& 0& 0\\ 0& 0& 0& 0& \bar{\Theta}_{45}& 0& 0& 0& 0\\ 0& 0& 0& 0& 0& 0& \bar{\Theta}_{57}& 0& 0 \end{array}\displaystyle \right ], \end{aligned}$$

where

$$\begin{gathered} \bar{\Theta}_{16}=-\sqrt{2}(R_{12}+R_{22}) \mathbb{C};\qquad \bar{\Theta}_{45} =\sqrt{2}(R_{11}+R_{21}) \mathbb{B};\qquad \bar{\Theta}_{57}=\sqrt {2}(R_{11}+R_{21})E, \\\Delta S_{2}(k)= \left [ \textstyle\begin{array}{c@{\quad}c@{\quad}c@{\quad}c@{\quad}c@{\quad }c@{\quad}c@{\quad}c@{\quad}c} 0& 0& 0& 0& 0& \kappa_{16}& 0& 0& 0\\ 0& 0& 0& 0& 0& 0& 0& 0& 0\\ 0& 0& 0& 0& 0& 0& 0& 0& 0\\ 0& 0& 0& 0& \kappa_{45}& 0& 0& 0& 0\\ 0& 0& 0& 0& 0& 0& \kappa_{57}& 0& 0 \end{array}\displaystyle \right ],\end{gathered} $$

where $\kappa_{16}=-\sqrt{2}(R_{12}+R_{22})\Delta\mathbb{C}(k)$, $\kappa_{45}=\sqrt{2}(R_{11}+R_{21})\Delta\mathbb{B}(k)$, $\kappa _{57}=\sqrt{2}(R_{11}+R_{21})\Delta E(k)$.

From Assumption 1, it follows readily that

25 $$ \Delta S_{1}(k)=\bar{T}_{1}N(k)\bar{W}_{1},\qquad \Delta S_{2}(k)=\bar {T}_{2}N(k)\bar{W}_{2}, $$

where

$$\begin{gathered} \bar{T_{1}}= \left [ \textstyle\begin{array}{c@{\quad}c@{\quad}c@{\quad}c@{\quad}c@{\quad }c@{\quad}c} 0& 0& 0& 0& -\sqrt{2}(R_{11}+R_{21})T& 0& 0\\ 0& 0& 0& 0& 0& 0& 0\\ 0& 0& 0& 0& 0& 0& 0\\ 0& 0& 0& \sqrt{2}(R_{12}+R_{22})T& 0& 0& 0\\ 0& 0& 0& 0& 0& \sqrt{2}(R_{12}+R_{22})T& 0 \end{array}\displaystyle \right ], \\\bar{T_{2}}= \left [ \textstyle\begin{array}{c@{\quad}c@{\quad}c@{\quad}c@{\quad}c@{\quad }c@{\quad}c@{\quad}c@{\quad}c} 0& 0& 0& 0& 0& -\sqrt{2}(R_{12}+R_{22})T& 0& 0& 0\\ 0& 0& 0& 0& 0& 0& 0& 0& 0\\ 0& 0& 0& 0& 0& 0& 0& 0& 0\\ 0& 0& 0& 0& \sqrt{2}(R_{11}+R_{21})T& 0& 0& 0& 0\\ 0& 0& 0& 0& 0& 0& \sqrt{2}(R_{11}+R_{21})T& 0& 0 \end{array}\displaystyle \right ], \\\bar{W_{1}}= \left [ \textstyle\begin{array}{c@{\quad}c@{\quad}c@{\quad}c@{\quad}c@{\quad }c@{\quad}c} 0& 0& 0& 0& W_{1}& 0& 0\\ 0& 0& 0& 0& 0& 0& 0\\ 0& 0& 0& 0& 0& 0& 0\\ 0& 0& 0& W_{4}& 0& 0& 0\\ 0& 0& 0& 0& 0& W_{6}& 0 \end{array}\displaystyle \right ],\\ \bar{W_{2}}= \left [ \textstyle\begin{array}{c@{\quad}c@{\quad}c@{\quad}c@{\quad}c@{\quad }c@{\quad}c@{\quad}c@{\quad}c} 0& 0& 0& 0& 0& W_{3}& 0& 0& 0\\ 0& 0& 0& 0& 0& 0& 0& 0& 0\\ 0& 0& 0& 0& 0& 0& 0& 0& 0\\ 0& 0& 0& 0& W_{2}& 0& 0& 0& 0\\ 0& 0& 0& 0& 0& 0& W_{5}& 0& 0 \end{array}\displaystyle \right ].\end{gathered} $$

Note that $\Lambda_{3}(k)$ and $\Lambda_{4}(k)$ can be decomposed as follows:

26 $$ \Lambda_{3}(k)=\Lambda_{3}+\Delta\Lambda_{3}(k) ,\qquad \Lambda _{4}(k)=\Lambda_{4}+\Delta\Lambda_{4}(k), $$

where

$$\begin{gathered} \Lambda_{3}= \left [ \textstyle\begin{array}{c@{\quad}c} \hat{\Lambda}_{11}& \ast\\ S_{1}& J_{1} \end{array}\displaystyle \right ]< 0,\qquad \Delta\Lambda_{3}(k)= \left [ \textstyle\begin{array}{c@{\quad}c} 0& \ast\\ \Delta S_{1}(k)& 0 \end{array}\displaystyle \right ], \\\Lambda_{4}= \left [ \textstyle\begin{array}{c@{\quad}c} \hat{\Lambda}_{22}& \ast\\ S_{2}& J_{2} \end{array}\displaystyle \right ]< 0 \quad\text{and} \quad\Delta\Lambda_{4}(k)= \left [ \textstyle\begin{array}{c@{\quad}c} 0& \ast\\ \Delta S_{2}(k)& 0 \end{array}\displaystyle \right ].\end{gathered} $$

Let

$$\begin{gathered} \tilde{T}_{1}^{T}=\bigl[0,\bar{T}_{1}^{T} \bigr], \quad\quad\tilde{W}_{1}=[\bar {W}_{1},0], \\ \tilde{T}_{2}^{T}=\bigl[0,\bar{T}_{2}^{T} \bigr], \qquad\tilde{W}_{2}=[\bar{W}_{2},0].\end{gathered} $$

Using Lemma 2.3(i), $\Delta\Lambda_{3}(k)$ and $\Delta\Lambda _{4}(k)$ can be rewritten as

27 $$\begin{aligned}& \Delta\Lambda_{3}(k)=\tilde{T}_{1}N(k) \tilde{W}_{1}+\tilde {W}_{1}^{T}N^{T}(k) \tilde{T}_{1}^{T}\leq\varepsilon_{1}^{-1} \tilde {T}_{1}\tilde{T}_{1}^{T} + \varepsilon_{1}\tilde{W}_{1}^{T} \tilde{W}_{1}, \\& \Delta\Lambda_{4}(k)=\tilde{T}_{2}N(k) \tilde{W}_{2}+\tilde {W}_{2}^{T}N^{T}(k) \tilde{T}_{2}^{T}\leq\varepsilon_{2}^{-1} \tilde {T}_{2}\tilde{T}_{2}^{T} + \varepsilon_{2}\tilde{W}_{2}^{T} \tilde{W}_{2}. \end{aligned}$$

It is clear from equations (26) and (27) that

28 $$ \Lambda_{3}(k)\leq\Lambda'_{3}+ \varepsilon_{1}^{-1}\tilde {T}_{1} \tilde{T}_{1}^{T},\qquad \Lambda_{4}(k)\leq \Lambda'_{4}+\varepsilon_{2}^{-1} \tilde {T}_{2}\tilde{T}_{2}^{T}, $$

where

$$\begin{gathered} \Lambda'_{3}= \left [ \textstyle\begin{array}{c@{\quad}c} \Lambda'_{11}+\mu_{1}\left [ {\scriptsize\begin{matrix}{} I_{2n\times2n}& 0\cr 0& 0 \end{matrix}} \right ]& \ast \\ S_{1}& J_{1} \end{array}\displaystyle \right ], \\\Lambda'_{4}= \left [ \textstyle\begin{array}{c@{\quad}c} \Lambda'_{22}+\mu_{2}\left [ {\scriptsize\begin{matrix}{} I_{2n\times2n}& 0\cr 0& 0 \end{matrix}} \right ]& \ast \\ S_{2}& J_{2} \end{array}\displaystyle \right ].\end{gathered} $$

It follows from Lemma 2.4 that equation (22) is equivalent to the case that the right-hand side of equation (28) is negative definite. Hence, we come to the conclusion that $\Lambda_{3}(k)<0$ and $\Lambda_{4}(k)<0$, and therefore equation (22) holds. Moreover, the combination of equations (20) and (22) leads to

29 $$ \mathbb{E}\bigl\{ \Delta\mathbb{V}(k)\bigr\} \leq\mu_{1}\mathbb{E} \bigl\{ \big|\bar {x}(k)\big|^{2}\bigr\} -\mu_{2}\mathbb{E}\bigl\{ \big| \bar{y}(k)\big|^{2}\bigr\} . $$

We are in a position to prove the stability of system (8). First, from equation (10), it is easily verified that

30 $$\begin{aligned}[b] \mathbb{E}\bigl\{ \Delta V(k)\bigr\} \leq{}&\varepsilon_{11}\mathbb{E}\bigl\{ \big|\bar {x}(k)\big|^{2}\bigr\} +\varepsilon_{21}\sum _{i=k-\tau_{M}}^{k-1}\mathbb{E}\bigl\{ \big|\bar{x}(i)\big|^{2} \bigr\} \\&+\varepsilon_{12}\mathbb{E}\bigl\{ \big|\bar{y}(k)\big|^{2} \bigr\} +\varepsilon_{22} \sum_{i=k-\delta_{M}}^{k-1} \mathbb{E}\bigl\{ \big|\bar{y}(i)\big|^{2}\bigr\} , \end{aligned} $$

where

$$\begin{gathered} \varepsilon_{11}=\max\bigl\{ \lambda_{\max}(R_{11}), \lambda_{\max}(R_{21}), \lambda_{\max}(R_{52}) \bigr\} , \\ \varepsilon_{21}=(\tau_{M}-\tau_{m}+1) \bigl( \lambda_{\max }(R_{31})+\lambda_{\max}(R_{41}) \bigr), \\ \varepsilon_{12}=\max\bigl\{ \lambda_{\max}(R_{12}), \lambda_{\max }(R_{22}),\lambda_{\max}(R_{51}) \bigr\} , \\ \varepsilon_{22}=(\delta_{M}-\delta_{m}+1) \bigl(\lambda_{\max }(R_{32})+\lambda_{\max}(R_{42}) \bigr).\end{gathered} $$

For any scalar $\zeta>1$, the above inequality, combined with equation (29), indicates that

31 $$\begin{aligned}& \begin{aligned}\zeta^{k+1}\mathbb{E}\bigl\{ \mathbb{V}(k+1)\bigr\} -\zeta^{k} \mathbb {E}\bigl(\mathbb{V}(k)\bigr)&=\zeta^{k+1}\mathbb{E}\bigl\{ \Delta \mathbb{V}(k)\bigr\} +\zeta^{k}(\zeta-1)\mathbb{E}\bigl\{ \mathbb{V}(k) \bigr\} \\ &\leq-\zeta^{k+1}\bigl(\mu_{1}\mathbb{E}\bigl\{ \big| \bar{x}(k)\big|^{2}\bigr\} - \mu_{2}\mathbb{E}\bigl\{ \big| \bar{y}(k)\big|^{2}\bigr\} \bigr)+\zeta^{k}(\zeta-1),\end{aligned} \\& \begin{aligned}[b]&\Biggl(\varepsilon_{11}\mathbb{E}\bigl\{ \big|\bar{x}(k)\big|^{2} \bigr\} +\varepsilon_{21} \sum_{i=k-\tau_{M}}^{k-1} \mathbb{E}\bigl\{ \big|\bar{x}(i)\big|^{2}\bigr\} +\varepsilon_{12} \mathbb{E}\bigl\{ \big|\bar{y}(k)\big|^{2}\bigr\} +\varepsilon _{22} \mathbb{E}\bigl\{ \big|\bar{y}(i)\big|^{2}\bigr\} \Biggr)\\&\quad=\zeta^{k} \eta_{11}(\zeta )\mathbb{E}\bigl\{ \big|\bar{x}(k)\big|^{2}\bigr\} +\zeta^{k}\eta_{21}(\zeta)\sum_{i=k-\tau_{M}}^{k-1} \mathbb{E}\bigl\{ \big|\bar{x}(i)\big|^{2}\bigr\} \\ &\qquad +\zeta^{k} \eta_{12}(\zeta)\mathbb{E}\bigl\{ \big|\bar{y}(k)\big|^{2}\bigr\} +\zeta ^{k}\eta_{22} (\zeta)\sum_{i=k-\delta_{M}}^{k-1} \mathbb{E}\bigl\{ \big|\bar{y}(i)\big|^{2}\bigr\} , \end{aligned} \end{aligned}$$

where

$$\begin{gathered} \eta_{11}(\zeta)=-\zeta\mu_{1}+(\zeta-1) \varepsilon_{11},\qquad \eta _{21}(\zeta)=(\zeta-1) \varepsilon_{21}, \\ \eta_{12}(\zeta)=-\zeta\mu_{2}+(\zeta-1) \varepsilon_{12} \quad\text{and} \quad\eta_{22}(\zeta)=(\zeta-1) \varepsilon_{22}.\end{gathered} $$

In addition, for any integer $N\geq\max\{\delta_{M},\tau_{M}\}+1$, summing both sides of equation (31) from 0 to $N-1$ with respect to *k*, we have

32 $$\begin{aligned}[b] &\zeta^{N}\mathbb{E}\bigl\{ \mathbb{V}(N)\bigr\} -\mathbb{E}\bigl\{ \mathbb{V}(0)\bigr\} \\&\quad\leq\eta_{11}(\zeta)\sum _{k=0}^{N-1}\zeta^{k}\mathbb{E}\bigl\{ \big|\bar {x}(k)\big|^{2}\bigr\} +\eta_{21}(\zeta)\sum _{k=0}^{N-1}\sum_{i=k-\tau_{M}}^{k-1} \zeta ^{k}\mathbb{E}\bigl\{ \big|\bar{x}(i)\big|^{2}\bigr\} \\ &\qquad+\eta_{12}(\zeta)\sum_{k=0}^{N-1} \zeta^{k}\mathbb{E}\bigl\{ \big|\bar{y}(k)\big|^{2}\bigr\} + \eta_{22}(\zeta)\sum_{k=0}^{N-1}\sum _{i=k-\delta_{M}}^{k-1}\zeta ^{k}\mathbb{E} \bigl\{ \big|\bar{y}(i)\big|^{2}\bigr\} .\end{aligned} $$

Note that, for $\tau_{M},\delta_{M}\geq1$,

33 $$\begin{aligned}& \sum_{k=0}^{N-1}\sum _{i=k-\tau_{M}}^{k-1}\zeta^{k}\mathbb{E}\bigl\{ \big| \bar{x}(i)\big|^{2}\bigr\} \leq\tau_{M}\zeta^{\tau_{M}}\max _{-\tau _{M}\leq i\leq0}\mathbb{E}\bigl\{ \big|\Omega(i)\big|^{2}\bigr\} + \tau_{M}\zeta^{\tau_{M}} \sum_{i=0}^{N-1} \zeta^{i}\mathbb{E}\bigl\{ \big|\bar{x}(k)\big|^{2}\bigr\} , \\& \sum_{k=0}^{N-1}\sum _{i=k-\delta_{M}}^{k-1}\zeta^{k} \mathbb{E}\bigl\{ \big| \bar{y}(i)\big|^{2}\bigr\} \leq\delta_{M}\zeta^{\delta _{M}} \max_{-\delta_{M}\leq i\leq0}\mathbb{E}\bigl\{ \big|\Pi(i)\big|^{2}\bigr\} + \delta_{M}\zeta^{\delta_{M}} \sum_{i=0}^{N-1} \zeta^{i}\mathbb{E}\bigl\{ \big|\bar{y}(k)\big|^{2}\bigr\} . \end{aligned}$$

Then, from equations (32) and (33), one has

34 $$\begin{aligned}[b] \zeta^{N}\mathbb{E}\bigl\{ \mathbb{V}(N)\bigr\} \leq{}&\mathbb{E}\bigl\{ \mathbb {V}(0)\bigr\} +\bigl[\eta_{11}(\zeta)+\tau_{M} \zeta^{\tau_{M}}\eta _{21}(\zeta)\bigr]\sum _{k=0}^{N-1}\zeta^{k}\mathbb{E}\bigl\{ \big|\bar {x}(k)\big|^{2}\bigr\} \\ &+\tau_{M}\zeta^{\tau_{M}}\eta_{21}(\zeta)\max _{-\tau _{M}\leq i\leq0}\mathbb{E}\bigl\{ \big|\Omega(i)\big|^{2}\bigr\} \\ &+\bigl[ \eta_{12}(\zeta )+\delta_{M}\zeta^{\delta_{M}} \eta_{22}(\zeta)\bigr]\sum_{k=0}^{N-1} \zeta^{k}\mathbb{E}\bigl\{ \big|\bar {y}(k)\big|^{2}\bigr\} \\ &+ \delta_{M}\zeta^{\delta_{M}}\eta_{22}(\zeta)\max _{-\delta_{M}\leq i\leq0}\mathbb{E}\bigl\{ \big|\Pi(i)\big|^{2}\bigr\} .\end{aligned} $$

Let

$$\begin{gathered} \varepsilon_{01}=\min\bigl\{ \lambda_{\min}(R_{11}), \lambda_{\min }(R_{21}) ,\lambda_{\min}(R_{52}) \bigr\} ,\qquad \tilde{\varepsilon}_{1}=\max\{ \varepsilon_{11}, \varepsilon_{21}\}, \\ \varepsilon_{02}=\min\bigl\{ \lambda_{\min}(R_{12}), \lambda_{\min }(R_{22}) ,\lambda_{\min}(R_{51}) \bigr\} ,\qquad \tilde{\varepsilon}_{2}=\max\{ \varepsilon_{12}, \varepsilon_{22}\}.\end{gathered} $$

It is clear that

35 $$ \mathbb{E}\bigl\{ \mathbb{V}(N)\bigr\} \geq\varepsilon_{01}\mathbb{E} \bigl\{ \big|\bar {x}(N)\big|^{2}\bigr\} +\varepsilon_{02}\mathbb{E} \bigl\{ \big|\bar{y}(N)\big|^{2}\bigr\} . $$

It follows readily from equation (30) that

36 $$ \mathbb{E}\bigl\{ \mathbb{V}(0)\bigr\} \leq\tilde{\varepsilon}_{1}\max _{-\tau _{M}\leq i\leq0}\mathbb{E}\bigl\{ \big|\Omega(i)\big|^{2}\bigr\} +\tilde{ \varepsilon }_{2}\max_{-\delta_{M}\leq i\leq0}\mathbb{E}\bigl\{ \big| \Pi(i)\big|^{2}\bigr\} . $$

Additionally, it can be verified that there exists a scalar $\zeta _{0}>1$ such that

37 $$\begin{aligned}& \eta_{11}(\zeta_{0})+\tau_{M} \zeta_{0}^{\tau_{M}}\eta_{21}(\zeta _{0})=0, \\ & \eta_{12}(\zeta_{0})+\delta_{M} \zeta_{0}^{\delta_{M}}\eta _{22}(\zeta_{0})=0. \end{aligned}$$

Substituting equations (35)–(37) into equation (34), we can get

38 $$\begin{aligned}[b] \varepsilon_{01}\mathbb{E}\bigl\{ \big|\bar{x}(N)\big|^{2}\bigr\} + \varepsilon _{02}\mathbb{E}\bigl\{ \big|\bar{y}(N)\big|^{2}\bigr\} \leq{}&\bigl(\tilde{\varepsilon }_{1}+\tau_{M} \zeta_{0}^{\tau_{M}}\eta_{21}(\zeta_{0})\bigr) \max_{-\tau_{M}\leq i\leq0}\mathbb{E}\bigl\{ \big|\Omega(i)\big|^{2}\bigr\} \\ &+\bigl(\tilde{\varepsilon}_{2}+\delta_{M} \zeta_{0}^{\delta _{M}}\eta_{22}(\zeta_{0})\bigr) \max_{-\delta_{M}\leq i\leq0}\mathbb {E}\bigl\{ \big|\Pi(i)\big|^{2}\bigr\} .\end{aligned} $$

The above equation (38) completes the proof of exponential stability with $v_{x}(k)=0$ and $v_{y}(k)=0$. □

### Remark 3.1

In this paper, we have considered the time-varying delays $\delta(k)$, $\tau(k)$ and the leakage delays $\rho_{1}$, $\rho_{2}$ in the negative feedback term of the GRNs which lead to the instability of the systems with small amount of leakage delay. This paper is to establish techniques to accord with the robust $H_{\infty}$ state estimation concern for uncertain discrete stochastic GRNs (equation (1)) with leakage delays, distributed delays, and probabilistic measurement delays.

Consider that the $H_{\infty}$ attainment of the estimation error system (8) is robustly stochastically stable with non-zero exogenous disturbance signals $v_{x}(k), v_{y}(k)\in L_{2} [0,\infty)$.

### Theorem 3.2

*Let Assumptions* 1 *and* 2 *hold*. *Let the leakage delays* $\rho_{1}$, $\rho_{2}$ *and the estimation parameters* $\mathbb {A}_{x}$, $\mathbb{B}_{x}$, $\mathbb{A}_{y}$, $\mathbb{B}_{y}$, *and* $\gamma>0$ *be given*. *Then the estimation error system* (8) *is robustly stochastically stable with disturbance attenuation* *γ*, *if there exist positive definite matrices* $R_{11}$, $R_{12}$, $R_{21}$, $R_{22}$, $R_{31}$, $R_{32}$, $R_{41}$, $R_{42}$, $R_{51}$, $R_{52}$ *and three positive constant scalars* *λ*, $\varepsilon_{1}$, *and* $\varepsilon_{2}$ *such that the following LMI holds*:

39 $$ \Lambda_{1}= \left [ \textstyle\begin{array}{c@{\quad}c@{\quad}c@{\quad}c} \Lambda'_{11}& \ast& \ast& \ast\\ 0& -\gamma^{2}I& \ast& \ast\\ S_{1}& 0& J_{1}& \ast\\ 0& 0& \bar{T}_{1}^{T}& -\varepsilon_{1}I \end{array}\displaystyle \right ]< 0,\qquad \Lambda_{2}= \left [ \textstyle\begin{array}{c@{\quad}c@{\quad}c@{\quad}c} \Lambda'_{22}& \ast& \ast& \ast\\ 0& -\gamma^{2}I& \ast& \ast\\ S_{2}& 0& J_{2}& \ast\\ 0& 0& \bar{T}_{2}^{T}& -\varepsilon_{2}I \end{array}\displaystyle \right ]< 0, $$

*and the other variables are described in Theorem *3.1.

### Proof

Choose the Lyapunov–Krasovskii function (equation (10)) as in Theorem 3.1. For given $\gamma>0$, we define

40 $$ T(n)=\mathbb{E}\sum_{k=0}^{n}\bigl[ \bar{x}^{T}(k)\bar{x}(k)+\bar {y}^{T}(k)\bar{y} (k)- \gamma^{2}v_{x}^{T}(k)v_{x}(k)- \gamma^{2}v_{y}^{T}(k)v_{y}(k)\bigr]. $$

Here, *n* is a nonnegative integer. Our aim is to show $T(n)<0$. Under the zero initial condition, we have

41 $$\begin{aligned}[b] T(n)&=\mathbb{E}\sum_{k=0}^{n}\bigl[ \bar{x}^{T}(k)\bar{x}(k)+\bar {y}^{T}(k)\bar{y}(k)- \gamma^{2}v_{x}^{T}(k)v_{x}(k)-\gamma ^{2}v_{y}^{T}(k)v_{y}(k)+\Delta \mathbb{V}(k)\bigr]\\ &\quad-\mathbb{E}\mathbb {V}(n+1) \\ &\leq T(n)+\sum_{k=0}^{n}\mathbb{E}\bigl( \Delta\mathbb{V}(k)\bigr) \\ &=\sum_{k=0}^{n}\mathbb{E}\bigl\{ \varpi^{T}(k)\bigl[\tilde{\Lambda }_{11}+\sigma_{\alpha} \tilde{W}_{01}^{T}R_{21}\tilde{W}_{01} +2 \tilde{G}_{01}^{T}(k) (R_{11}+R_{21}) \tilde{G}_{01}(k) \\ &\quad+2\tilde{F}_{01}^{T}(k)R_{21} \tilde{F}_{01}(k)+2\tilde {G}_{11}^{T}(k) (R_{12}+R_{22})\tilde{G}_{11}(k) \\ &\quad+2\tilde{S}_{01}^{T}(k) (R_{12}+R_{22}) \tilde {S}_{01}(k)\bigr]\varpi(k)\\&\quad +\Gamma^{T}(k)\bigl[\tilde{ \Lambda}_{22}+\sigma_{\beta}\tilde {W}_{02}^{T}R_{22} \tilde{W}_{02}+2\tilde{G}_{02}^{T}(k) (R_{12}+R_{22}) \tilde {G}_{02}(k)\\ &\quad+2\tilde{F}_{02}^{T}(k)R_{22} \tilde{F}_{02}(k) +2\tilde{G}_{12}^{T}(k) (R_{11}+R_{21}) \tilde {G}_{12}(k)\\ &\quad+2\tilde{S}_{02}^{T}(k) (R_{11}+R_{21})\tilde {S}_{02}(k)\bigr]\Gamma(k) \bigr\} ,\end{aligned} $$

where

$$\begin{gathered} \varpi(k)=\bigl[\varpi_{0}(k),v_{x}(k) \bigr]^{T},\qquad \Gamma(k)=\bigl[\Gamma _{0}(k),v_{y}(k) \bigr]^{T}, \qquad\tilde{W}_{01}=\bigl[\hat{W}_{01}^{T},0 \bigr], \\ \tilde{G}_{01}(k)=\bigl[\hat{G}_{01}^{T}(k),0 \bigr], \qquad\tilde{F}_{01}(k)=\bigl[\hat {F}_{01}^{T}(k),0 \bigr], \qquad\tilde{G}_{11}(k)=\bigl[\hat{G}_{11}^{T}(k),0 \bigr], \\ \tilde{W}_{02}=\bigl[\hat{W}_{02}^{T},0\bigr],\qquad \tilde{G}_{02}(k)=\bigl[\hat {G}_{02}^{T}(k),0 \bigr], \qquad\tilde{F}_{02}(k)=\bigl[\hat{F}_{02}^{T}(k),0 \bigr], \\ \tilde{G}_{12}(k)=\bigl[\hat{G}_{12}^{T}(k),0 \bigr], \qquad\tilde{S}_{01}(k)=\bigl[\hat {S}_{01}^{T}(k),0 \bigr], \qquad\tilde{S}_{02}(k)=\bigl[\hat{S}_{02}^{T}(k),0 \bigr], \\\tilde{\Lambda}_{11}= \left [ \textstyle\begin{array}{c@{\quad}c} \Lambda_{11}& 0\\ 0& -\gamma^{2}I \end{array}\displaystyle \right ] \quad\text{and} \quad\tilde{\Lambda}_{22}= \left [ \textstyle\begin{array}{c@{\quad}c} \Lambda_{22}& 0\\ 0& -\gamma^{2}I \end{array}\displaystyle \right ].\end{gathered} $$

By equation (41), in order to assure $T(n)<0$, we just need to show

42 $$\begin{aligned}& \tilde{\Lambda}_{11}+\sigma_{\alpha}\tilde{W}_{01}^{T}R_{21} \tilde {W}_{01}+2\tilde{G}_{01}^{T}(k) (R_{11}+R_{21})\tilde{G}_{01}(k) +2 \tilde{F}_{01}^{T}(k)R_{21}\tilde{F}_{01}(k) \\& \quad+2\tilde{G}_{11}^{T}(k) (R_{12}+R_{22}) \tilde{G}_{11}(k) +2\tilde{S}_{01}^{T}(k) (R_{12}+R_{22})\tilde{S}_{01}(k)< 0, \\& \tilde{\Lambda}_{22}+\sigma_{\beta}\tilde{W}_{02}^{T}R_{22} \tilde {W}_{02}+2\tilde{G}_{02}^{T}(k) (R_{12}+R_{22})\tilde {G}_{02}(k)+2 \tilde{F}_{02}^{T}(k)R_{22}\tilde{F}_{02}(k) \\& \quad+2\tilde{G}_{12}^{T}(k) (R_{11}+R_{21}) \tilde{G}_{12}(k)+2\tilde {S}_{02}^{T}(k) (R_{11}+R_{21})\tilde{S}_{02}(k)< 0, \end{aligned}$$

which, by Lemma 2.4, is equivalent to

43 $$ \tilde{\Lambda}_{3}(k)= \left [ \textstyle\begin{array}{c@{\quad}c} \tilde{\Lambda}_{11}& \ast\\ \bar{S}_{1}(k)& J_{1} \end{array}\displaystyle \right ]< 0\quad \text{and}\quad \tilde{\Lambda}_{4}(k)= \left [ \textstyle\begin{array}{c@{\quad}c} \Lambda_{22}& \ast\\ \bar{S}_{2}(k)& J_{2} \end{array}\displaystyle \right ]< 0, $$

where

$$\begin{gathered} \bar{S}_{1}(k)=\bar{S}_{1}+\Delta\bar{S}_{1}(k)=[S_{1},0]+ \bigl[\Delta S_{1}(k),0\bigr], \\ \bar{S}_{2}(k)=\bar{S}_{2}+\Delta\bar{S}_{2}(k)=[S_{2},0]+ \bigl[\Delta S_{2}(k),0\bigr]\end{gathered} $$

and $J_{1}$ and $J_{2}$ are defined in Theorem 3.1. Note that $\tilde{\Lambda}_{3}(k)$ and $\tilde{\Lambda}_{4}(k)$ can be rearranged as follows:

44 $$ \tilde{\Lambda}_{3}(k)=\tilde{\Lambda}_{3}+\Delta\tilde{ \Lambda }_{3}(k), \quad\quad\tilde{\Lambda}_{4}(k)=\tilde{ \Lambda}_{4}+\Delta\tilde {\Lambda}_{4}(k), $$

where

$$\begin{gathered} \tilde{\Lambda}_{3}= \left [ \textstyle\begin{array}{c@{\quad}c} \tilde{\Lambda}_{11}& \ast\\ \bar{S}_{1}& J_{1} \end{array}\displaystyle \right ]< 0 \quad\text{and} \quad\Delta\tilde{\Lambda}_{3}(k)= \left [ \textstyle\begin{array}{c@{\quad}c} 0& \ast\\ \Delta\bar{S}_{1}(k)& 0 \end{array}\displaystyle \right ], \\ \tilde{\Lambda}_{4}= \left [ \textstyle\begin{array}{c@{\quad}c} \tilde{\Lambda}_{22}& \ast\\ \bar{S}_{2}& J_{2} \end{array}\displaystyle \right ]< 0 \quad\text{and} \quad\Delta\tilde{\Lambda}_{4}(k)= \left [ \textstyle\begin{array}{c@{\quad}c} 0& \ast\\ \Delta\bar{S}_{2}(k)& 0 \end{array}\displaystyle \right ].\end{gathered} $$

Let

$$\begin{gathered} \breve{T}_{1}^{T}=\bigl[0,\tilde{T}_{1}^{T} \bigr], \qquad\breve{W}_{1}=[\tilde {W}_{1},0],\qquad \breve{T}_{2}^{T}=\bigl[0,\tilde{T}_{2}^{T} \bigr], \qquad\breve {W}_{2}=\bigl[\tilde{W}_{2}^{T},0 \bigr], \\ \breve{T}_{1}^{T}=\bigl[0,0,\bar{T}_{1}^{T} \bigr], \qquad\breve{W}_{1}=[\bar {W}_{1},0,0],\qquad \breve{T}_{2}^{T}=\bigl[0,0,\bar{T}_{2}^{T} \bigr] \quad\text{and} \quad\breve {W}_{2}=[\bar{W}_{2},0,0].\end{gathered} $$

Using Lemma 2.3(i), $\Delta\Lambda_{3}(k)$ and $\Delta\Lambda _{4}(k)$ can be rewritten as

45 $$\begin{aligned}& \Delta\tilde{\Lambda}_{3}(k)=\breve{T}_{1}N(k)\breve {W}_{1}+\breve{W}_{1}^{T}N^{T}(k) \breve{T}_{1}^{T}\leq\epsilon _{1}^{-1} \breve{T}_{1}\breve{T}_{1}^{T} + \epsilon_{1}\breve{W}_{1}^{T}\breve{W}_{1}, \\& \Delta\tilde{\Lambda}_{4}(k)=\breve{T}_{2}N(k)\breve {W}_{2}+\breve{W}_{2}^{T}N^{T}(k) \breve{T}_{2}^{T}\leq\epsilon _{2}^{-1} \breve{T}_{2}\breve{T}_{2}^{T} + \epsilon_{1}\breve{W}_{2}^{T}\breve{W}_{2}. \end{aligned}$$

It is implied from equations (44) and (45) that

46 $$\begin{gathered} \tilde{\Lambda}_{3}(k)\leq \left [ \textstyle\begin{array}{c@{\quad}c@{\quad}c} \Lambda'_{11}& \ast& \ast\\ 0& -\gamma^{2}I& \ast\\ S_{1}& 0& J_{1} \end{array}\displaystyle \right ]+\epsilon_{1}^{-1}\breve{T}_{1} \breve{T}_{1}^{T}, \\\tilde{\Lambda}_{4}(k)\leq \left [ \textstyle\begin{array}{c@{\quad}c@{\quad}c} \Lambda'_{22}& \ast& \ast\\ 0& -\gamma^{2}I& \ast\\ S_{2}& 0& J_{2} \end{array}\displaystyle \right ]+\epsilon_{2}^{-1} \breve{T}_{2}\breve{T}_{2}^{T}.\end{gathered} $$

Using Lemma 2.4, the above inequality (45) holds if and only if the right-hand side of (45) is negative definite, which implies $T(n)<0$. Letting $n\rightarrow\infty$, we have

$$ \mathbb{E}\sum_{k=0}^{\infty}\bigl\{ \big| \bar{x}(k)\big|^{2}+\big|\bar{y}(k)\big|^{2}\bigr\} \leq \gamma^{2}\mathbb{E}\sum_{k=0}^{\infty} \bigl(\big|v_{x}(k)\big|^{2}+\big|v_{y}(k)\big|^{2} \bigr). $$

Hence the proof of Theorem 3.2 is complete. □

### Theorem 3.3

*With the help of the assumptions*, *system* (7) *becomes a robust* $H_{\infty}$ *state estimator of GRNs* (1) *with leakage delays*, *distributed delays*, *and probabilistic measurement delays* (5) *if there exist positive definite matrices* $X_{1}$, $X_{2}$, $Y_{1}$, $Y_{2}$, $R_{11}$, $R_{12}$, $R_{21}$, $R_{22}$, $R_{31}$, $R_{32}$, $R_{41}$, $R_{42}$, $R_{51}$, *and* $R_{52}$ *and three positive constant scalars* *λ*, $\varepsilon_{1}$, *and* $\varepsilon_{2}$ *such that the following LMIs hold*:

$$ \Lambda_{1}= \left [ \textstyle\begin{array}{c@{\quad}c@{\quad}c@{\quad}c} \Lambda'_{11}& \ast& \ast& \ast\\ 0& -\gamma^{2}I& \ast& \ast\\ S'_{1}& 0& J_{1}& \ast\\ 0& 0& \bar{T}_{1}^{T}& -\varepsilon_{1}I \end{array}\displaystyle \right ]< 0,\qquad \Lambda_{2}= \left [ \textstyle\begin{array}{c@{\quad}c@{\quad}c@{\quad}c} \Lambda'_{22}& \ast& \ast& \ast\\ 0& -\gamma^{2}I& \ast& \ast\\ S'_{2}& 0& J_{2}& \ast\\ 0& 0& \bar{T}_{2}^{T}& -\varepsilon_{2}I \end{array}\displaystyle \right ]< 0, $$

*where*

$$\begin{gathered} S'_{1}= \left [ \textstyle\begin{array}{c@{\quad}c@{\quad}c@{\quad}c@{\quad}c@{\quad }c@{\quad}c} 0& 0& 0& 0& \Sigma_{15}& 0& 0\\ \sqrt{2}(X_{1}-\alpha_{0}X_{2}M)& -\sqrt{2}X_{1}& -\sqrt {2}(1-\alpha_{0})X_{2}M& 0& 0& 0& 0\\ \sqrt{\sigma_{\alpha}}X_{2}M& 0& \sqrt{\sigma_{\alpha}}X_{2}M& 0& 0& 0& 0\\ 0& 0& 0& \sqrt{2}(R_{12}+R_{22})\mathbb{D}& 0& 0& 0\\ 0& 0& 0& 0& 0& \Sigma_{56}& 0 \end{array}\displaystyle \right ], \\\Sigma_{15}=-\sqrt{2}(R_{11}+R_{21})A ;\qquad \Sigma_{56}=\sqrt {2}(R_{12}+R_{22})F, \\S'_{2}= \left [ \textstyle\begin{array}{c@{\quad}c@{\quad}c@{\quad}c@{\quad}c@{\quad }c@{\quad}c@{\quad}c@{\quad}c} 0& 0& 0& 0& 0& \Upsilon_{16}& 0& 0& 0\\ \sqrt{2}(Y_{1}-\beta_{0}Y_{2}N)& -\sqrt{2}Y_{1}& -\sqrt{2}(1-\beta _{0})Y_{2}N& 0& 0& 0& 0& 0& 0\\ \sqrt{\sigma_{\beta}}Y_{2}N& 0& \sqrt{\sigma_{\beta}}Y_{2}N& 0& 0& 0& 0& 0& 0\\ 0& 0& 0& 0& \sqrt{2}(R_{11}+R_{21})\mathbb{B}& 0& 0& 0& 0\\ 0& 0& 0& 0& 0& 0& \Upsilon_{57}& 0& 0 \end{array}\displaystyle \right ], \\\Upsilon_{16}=-\sqrt{2}(R_{12}+R_{22})\mathbb{C};\qquad \Upsilon _{57}=\sqrt{2}(R_{11}+R_{21})E,\end{gathered} $$

*and the other variables are described in Theorem *3.1. *Furthermore*, *the state estimator gain matrices can be described as follows*:

$$ \mathbb{A}_{x}=R_{21}^{-1}X_{1},\qquad \mathbb{B}_{x}=R_{21}^{-1}X_{2},\qquad \mathbb{A}_{y}=R_{22}^{-1}Y_{1} \quad\textit{and}\quad \mathbb{B}_{y}=R_{22}^{-1}Y_{2}. $$

### Proof

The rest of the proof of this theorem is the same as that of Theorem 3.2. Due to the limitation of the length of this paper, we omit it here. Then the proof of Theorem 3.3 is completed. □

Consider the discrete-time genetic regulatory network system:

47 $$\begin{aligned}& \begin{aligned}x(k+1)={}&{-}\mathbb{A}x(k-\rho_{1})+\mathbb{B} \hat{g}\bigl(y\bigl(k- \delta (k)\bigr)\bigr)+E\sum_{s=1}^{\infty} \mu_{s}h\bigl(y(k-s)\bigr)\\ &+\sigma\bigl(k,x(k-\rho _{1}) \bigr)\omega(k) +L_{x}v_{x}(k),\end{aligned} \\& y(k+1)=-\mathbb{C}y(k-\rho_{2})+\mathbb{D}x\bigl(k-\tau(k)\bigr)+F \sum_{n=1}^{\infty}\xi_{n}x(k-n)+L_{y}v_{y}(k). \end{aligned}$$

### Corollary 3.1

*Let the leakage delays* $\rho_{1}$, $\rho_{2}$ *and the estimation parameters* $\mathbb{A}_{x}$, $\mathbb{B}_{x}$, $\mathbb{A}_{y}$, *and* $\mathbb{B}_{y}$ *be given and also the acceptable conditions hold*. *Then the estimation error system* (8) *with* $v_{x}(k)=0$ *and* $v_{y}(k)=0$ *is robustly exponentially stable in the mean square if there exist positive definite matrices* $R_{11}$, $R_{12}$, $R_{21}$, $R_{22}$, $R_{31}$, $R_{32}$, $R_{41}$, $R_{42}$, $R_{51}$, $R_{52}$ *and the positive constant scalar* *λ* *such that the following LMI holds*:

48 $$ \Lambda_{1}= \left [ \textstyle\begin{array}{c@{\quad}c@{\quad}c} \Lambda'_{11}& \ast& \ast\\ S_{1}& J_{1}& \ast\\ 0& 0& I \end{array}\displaystyle \right ]< 0,\qquad \Lambda_{2}= \left [ \textstyle\begin{array}{c@{\quad}c@{\quad}c} \Lambda'_{22}& \ast& \ast\\ S_{2}& J_{2}& \ast\\ 0& 0& I \end{array}\displaystyle \right ]< 0, $$

*where*

$$\begin{gathered} \Lambda'_{11}= \left [ \textstyle\begin{array}{c@{\quad}c@{\quad}c@{\quad}c@{\quad}c@{\quad }c@{\quad}c} \psi_{11}& 0& 0& 0& 0& 0& 0 \\ 0& -R_{21}& 0& 0& 0& 0& 0\\ 0& 0& -R_{31}& 0& 0& 0& 0\\ 0& 0& 0& -R_{41}+\varepsilon_{1}W_{4}^{T}W_{4}& 0& 0& 0\\ 0& 0& 0& 0& HR_{11}& 0& 0\\ 0& 0& 0& 0& 0& I(R_{12}+R_{22})& 0\\ 0& 0& 0& 0& 0& 0& -\bar{\xi}R_{52} \end{array}\displaystyle \right ], \\\Lambda'_{22}= \left [ \textstyle\begin{array}{c@{\quad}c@{\quad}c@{\quad}c@{\quad}c@{\quad }c@{\quad}c@{\quad}c@{\quad}c} \psi_{12}& 0& 0& 0& 0& 0& 0& 0& 0 \\ 0& -R_{22}& 0& 0& 0& 0& 0& 0& 0\\ 0& 0& -R_{32}& 0& 0& 0& 0& 0& 0\\ 0& 0& 0& -R_{42}-\lambda\tilde{N}_{1}+\varepsilon_{2}W_{2}^{T}W_{2}& -\lambda\tilde{N}_{2}^{T}& 0& 0& 0& 0\\ 0& 0& 0& -\lambda\tilde{N}_{2}& -\lambda I& 0& 0& 0& 0\\ 0& 0& 0& 0& 0& 0& 0& 0& 0\\ 0& 0& 0& 0& 0& 0& I(R_{11}+R_{21})& 0& 0\\ 0& 0& 0& 0& 0& 0& 0& \bar{\mu}R_{51}& 0\\ 0& 0& 0& 0& 0& 0& 0& 0& -\bar{\mu}R_{51} \end{array}\displaystyle \right ], \\S_{1}= \left [ \textstyle\begin{array}{c@{\quad}c@{\quad}c@{\quad}c@{\quad}c@{\quad }c@{\quad}c} 0& 0& 0& 0& \bar{\Xi}_{15}& 0& 0\\ \Xi_{21}& -\sqrt{2}R_{21}\mathbb{A}_{x}& \Xi_{23}& 0& 0& 0& 0\\ \sqrt{\sigma_{\alpha}}R_{21}\mathbb{B}_{x}M& 0& \sqrt{\sigma _{\alpha}}R_{21}\mathbb{B}_{x}M& 0& 0& 0& 0\\ 0& 0& 0& \bar{\Xi}_{44}& 0& 0& 0\\ 0& 0& 0& 0& 0& \bar{\Xi}_{55}& 0 \end{array}\displaystyle \right ],\end{gathered} $$

*where*

$$\begin{gathered} \bar{\Xi}_{15}=-\sqrt{2}(R_{11}+R_{21}) \mathbb{A};\qquad \bar{\Xi}_{44}=\sqrt{2}(R_{12}+R_{22}) \mathbb{D};\qquad \bar{\Xi }_{55}=\sqrt{2}(R_{12}+R_{22})F; \\ \Xi_{21}=\sqrt{2}R_{21}(\mathbb{A}_{x}- \alpha_{0}\mathbb{B}_{x}M); \qquad\Xi_{23}=- \sqrt{2}R_{21}(1-\alpha_{0})\mathbb{B}_{x}M, \\S_{2}= \left [ \textstyle\begin{array}{c@{\quad}c@{\quad}c@{\quad}c@{\quad}c@{\quad }c@{\quad}c@{\quad}c@{\quad}c} 0& 0& 0& 0& 0& \bar{\Theta}_{16}& 0& 0& 0\\ \Theta_{21}& -\sqrt{2}R_{22}\mathbb{A}_{y}& \Theta_{23}& 0& 0& 0& 0& 0& 0\\ \sqrt{\sigma_{\beta}}R_{22}\mathbb{B}_{y}N& 0& \sqrt{\sigma _{\beta}}R_{22}\mathbb{B}_{y}N& 0& 0& 0& 0& 0& 0\\ 0& 0& 0& 0& \bar{\Theta}_{45}& 0& 0& 0& 0\\ 0& 0& 0& 0& 0& 0& \bar{\Theta}_{57}& 0& 0 \end{array}\displaystyle \right ],\end{gathered} $$

*where*

$$\begin{aligned}& \bar{\Theta}_{16}=-\sqrt{2}(R_{12}+R_{22}) \mathbb{C}; \quad\quad\bar{\Theta}_{45}=\sqrt{2}(R_{11}+R_{21}) \mathbb{B}; \quad\quad\bar{\Theta }_{57}=\sqrt{2}(R_{11}+R_{21})E; \\& \Theta_{21}=\sqrt{2}R_{22}(A_{y}- \beta_{0}\mathbb{B}_{y}N);\qquad \Theta_{23}=- \sqrt{2}R_{22} (1-\beta_{0})\mathbb{B}_{y}N, \\& J_{1}=\operatorname{diag}\bigl\{ -(R_{11}+R_{21}),-R_{21},-R_{21},-(R_{12}+R_{22}),-(R_{12}+R_{22}) \bigr\} , \\& J_{2}=\operatorname{diag}\bigl\{ -(R_{12}+R_{22}),-R_{22},-R_{22},-(R_{11}+R_{21}),-(R_{11}+R_{21}) \bigr\} , \\& \bar{\mu}=\sum_{s=1}^{\infty}\mu_{s},\qquad \bar{\xi}=\sum_{n=1}^{\infty}\xi_{n}, \\& \psi_{11}=-R_{11}+R_{31}+(\tau_{M}- \tau_{m}+1)R_{41}+\bar{\xi }R_{52};\qquad \psi_{12}=-R_{12}+R_{32}+(\delta_{M}- \delta_{m}+1)R_{42}, \\& \tilde{N}_{1}=\frac{(N_{1}^{T}N_{2}+N_{2}^{T}N_{1})}{2}; \quad\quad\tilde{N}_{2} =- \frac{(N_{1}^{T}+N_{2}^{T})}{2}. \end{aligned}$$

## Numerical examples

In this part, two mathematical examples with simulations are provided to show the effectiveness of the proposed robust state estimator.

### Example 4.1

Consider the discrete-time GRN (1) with parameters given as follows:

$$\begin{gathered} A= \begin{pmatrix} 0.1& 0\\ 0& 0.2 \end{pmatrix} ,\qquad B= \begin{pmatrix} 0.08& 0\\ 0& 0.2 \end{pmatrix} ,\\ C=D= \begin{pmatrix} 0.1& 0\\ 0& 0.1 \end{pmatrix} ,\qquad E= \begin{pmatrix} 0.36& 0\\ 0& 0.1 \end{pmatrix} , \\ d_{1}= \begin{pmatrix} 0.1& 0\\ 0& 0.1 \end{pmatrix} , \qquad d_{2}= \begin{pmatrix} 0.28& 0\\ 0& 0.135 \end{pmatrix} ,\\ L_{x}= \begin{pmatrix} 0.2& 0\\ 0& 0.5 \end{pmatrix} ,\qquad L_{y}= \begin{pmatrix} 0.5& 0\\ 0& 0.2 \end{pmatrix} , \\ W_{1}=W_{2}=W_{3}=W_{4}=W_{5}=W_{6}= \begin{pmatrix} 0.3& 0\\ 0& 0.3 \end{pmatrix} ,\qquad \mu=\xi=\exp(-2), \\ F=0.4I, \qquad R=0.2I,\qquad G= \begin{pmatrix} \sin(k)& 0\\ 0& \cos(k) \end{pmatrix} ,\end{gathered} $$

and the leakage delays $\rho_{1}=\rho_{2}=1$. The regulatory function is taken as $g(s)=\frac{s^{2}}{1+s^{2}}$. The time-varying delays are chosen as $\delta(k)=3+(2*\sin(k*\pi/2))$ and $\tau (k)=3+(2*\cos(k*\pi/2))$, and the exogenous disturbance inputs are selected as $v_{x}(k)=\sin(6k)\exp(-0.1k)$ and $v_{y}(k)=\cos(2k)\exp(-0.2k)$.

Now consider the estimation error system (8) with parameters given by

$$\begin{gathered} A=0.1I,\qquad B= \begin{pmatrix} -0.1& 0\\ 0& 0.2 \end{pmatrix} ,\qquad E=F=0.3I,\qquad C=D=0.2I, \\ M= \begin{pmatrix} 0.6& 0\\ 0& 0.1 \end{pmatrix} ,\qquad N= \begin{pmatrix} 0.4& 0\\ 0.3& 0.5 \end{pmatrix} ,\qquad R= \begin{pmatrix} 0.1& 0\\ 0& 0.3 \end{pmatrix} , \\ N(k)= \begin{pmatrix} \sin(k*\pi/2)& 0\\ 0& \cos(k*\pi/2) \end{pmatrix} ,\qquad \alpha=0.001,\qquad \beta=0.003, \\ d_{1}= \begin{pmatrix} 0.2\times(\cos(\pi/2)-2)& 0\\ 0& 0.1\times(\sin(\pi/2)-1) \end{pmatrix} , \qquad d_{2}= \begin{pmatrix} 0.28& 0\\ 0& 0.135 \end{pmatrix} , \\ L_{x}= \begin{pmatrix} 0.5& 0\\ 0& 0.2 \end{pmatrix} , \quad L_{y}= \begin{pmatrix} 0.1& 0\\ 0& 0.1 \end{pmatrix} , \\W_{1}=W_{2}=W_{3}=W_{4}=W_{5}=W_{6}=0.1I,\quad\quad \mu=\xi=\exp(-1), \end{gathered}$$

and the leakage delays $\rho_{1}=\rho_{2}=1$. The exogenous disturbance inputs are selected as

$$ v_{x}(k)=(\sin6k)\exp(-0.1k),\qquad v_{y}(k)=(\cos2k) \exp(-0.1k). $$

The regulatory function is taken as $g(s)=\frac{s^{2}}{1+s^{2}}$. The time-varying delays are chosen as $\delta(k)=3+(2*\sin(k*\pi/2))$ and $\tau(k)=3+(2*\cos(k*\pi/2))$. By using the Matlab LMI toolbox, LMIs (40) and (41) are solved and a set of feasible solutions is obtained as follows:

$$\begin{gathered} X_{1}= \begin{pmatrix} 0.4338& -0.0041\\ -0.0041& 0.2852 \end{pmatrix} , \qquad X_{2}= \begin{pmatrix} 0.0210& -0.0260\\ -0.0260& 0.0533 \end{pmatrix} ,\\ R_{11}= \begin{pmatrix} 9.3434& 0.0025\\ 0.0025& 6.9265 \end{pmatrix} , \quad\quad R_{21}= \begin{pmatrix} 0.2169& -0.4861\\ -0.4861& 0.9363 \end{pmatrix} , \\ Y_{1}= \begin{pmatrix} 1.1918& -0.0128\\ -0.0128& 0.5832 \end{pmatrix} ,\qquad Y_{2}= \begin{pmatrix} 1.0836& -0.2279\\ -0.2279& 0.1073 \end{pmatrix} . \end{gathered}$$

The state estimator gain matrices can be determined as follows:

$$\begin{gathered} A_{x}= \begin{pmatrix} 1.2173& 0.4060\\ 0.6324& 0.1804 \end{pmatrix} , \qquad A_{y}= \begin{pmatrix} 0.2203& 0.0032\\ 0.0063& 0.2096 \end{pmatrix} , \\ B_{x}= \begin{pmatrix} 2.1102& 0.4831\\ 1.3729& 0.3185 \end{pmatrix} , \qquad B_{y}= \begin{pmatrix} 0.2005& 0.4226\\ 0.8342& 0.3887 \end{pmatrix} .\end{gathered} $$

**Figure 1:**
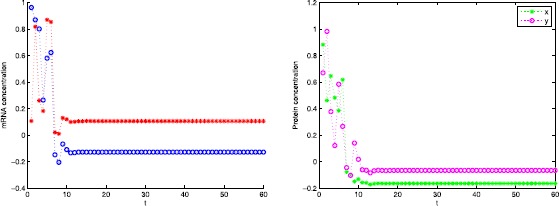
mRNA and protein concentration

**Figure 2:**
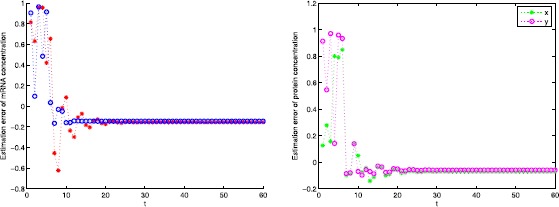
Estimation error for mRNA and protein concentration

The concentration of mRNA and protein and their estimation error are illustrated in Figs. 1 and 2 with the initial conditions $\phi _{1}(k)=\{1,0.1\}$, $\psi_{1}(k)=\{0.9,0.7\}$, $\phi_{2}(k)=\{ 0.9,0.8\}$, and $\psi_{2}(k)=\{0.15,0.9\}$.

### Example 4.2

**Figure 3:**
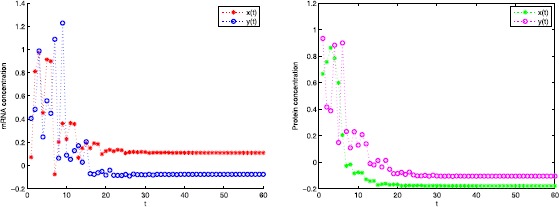
The state response $x(t)$, $y(t)$ of equation (47) with the mRNA and protein concentrations

Consider the discrete-time GRN (47) with parameters given by

$$\begin{aligned}& A= \begin{pmatrix} 0.3& 0\\ 0& 0.2 \end{pmatrix} ,\qquad B= \begin{pmatrix} -0.5& 0\\ 2.5& 0 \end{pmatrix} ,\qquad C= \begin{pmatrix} 0.1& 0\\ 0& 0.2 \end{pmatrix} ,\qquad D= \begin{pmatrix} 0.08& 0\\ 0& 0.2 \end{pmatrix} , \\& E= \begin{pmatrix} 0.36& 0\\ 0& 0.1 \end{pmatrix} ,\qquad F= \begin{pmatrix} 0.4& 0\\ 0& 0.4 \end{pmatrix} ,\\& d_{1}= \begin{pmatrix} 0.6& 0\\ 0& 0.1 \end{pmatrix} ,\qquad d_{2}= \begin{pmatrix} 0.28& 0\\ 0& 0.135 \end{pmatrix} , \\& L_{x}= \begin{pmatrix} 0.3& 0\\ 0& 0.4 \end{pmatrix} , \qquad L_{y}= \begin{pmatrix} 0.5& 0\\ 0& 0.2 \end{pmatrix} , \end{aligned}$$

and the leakage delays $\rho_{1}=\rho_{2}=1$. The regulatory function is taken as $g(s)=\frac{s^{2}}{1+s^{2}}$. The time-varying delays are chosen as $\delta(k)=2$ and $\tau(k)=1$, and the exogenous disturbance inputs are selected as $v_{x}(k)=\sin(6k)\exp(-0.1k)$ and $v_{y}(k)=\cos(2k)\exp(-0.2k)$. The the state responses $x(t)$ and $y(t)$ are shown in Fig. 3.

## Conclusions

In this paper, we have studied the approximation concern for the discrete-time stochastic GRNs with the leakage delays, distributed delays, and probabilistic measurement delays into the problem and modeled the robust $H_{\infty}$ state estimator for a class of discrete-time stochastic GRNs. Here, the probabilistic measurement delays, which narrate the binary shifting sequence, are satisfied by the conditional probability distribution. So, the crisis of parameter uncertainties, including errors, stochastic disturbance, leakage delays, distributed delays, and the activation function of the addressed GRNs, is identified by sector-bounded nonlinearities. By applying the Lyapunov stability theory and stochastic analysis techniques, sufficient conditions are first entrenched to assure the presence of the desired estimators in terms of a linear matrix inequality (LMI). These circumstances are reliant on both the lower and upper bounds of time-varying delays. Again, the absolute expression of the desired estimator is demonstrated to assure the estimation error dynamics to be robustly exponentially stable in the mean square for the consigned system. Lastly, numerical simulations have been utilized to illustrate the suitability and usefulness of our advanced theoretical results.
